# Evidence for regulation of transpiration in nonstomatal plants: insights from bryophyte gametophytes

**DOI:** 10.1111/nph.71206

**Published:** 2026-04-24

**Authors:** Alicia V. Perera‐Castro, Diego A. Márquez, Florian A. Busch, David T. Hanson

**Affiliations:** ^1^ Department of Biology Universitat de les Illes Balears Palma 07122 Spain; ^2^ University of New Mexico Albuquerque NM 87131 USA; ^3^ School of Biosciences University of Birmingham Birmingham B15 2TT UK; ^4^ Birmingham Institute of Forest Research University of Birmingham Birmingham B15 2TT UK

**Keywords:** bryophytes, capacitance, gas exchange, poikilohydry, pressure–volume, transpiration, water control

## Abstract

- Bryophyta (mosses) are a basal group of plants that lack stomata in their haploid form. Consequently, these plants are defined as poikilohydric, meaning poor control over water loss, and their cytosol is assumed to reach equilibrium with ambient humidity. This classification does not fully align with the diverse strategies observed in mosses.
- We studied gas exchange in 16 species under controlled dehydration conditions.
- Our results revealed that the cell wall water potential was below the cytosolic water potential in most species, as reflected by relative humidity at the cell wall surface decreasing to as low as 60% even under optimum hydric conditions. This indicates that cell membranes and/or cell walls involve barriers with higher resistance to water movement than previously assumed (2.7–23 MPa m^2^ s mmol^−1^). Additionally, species with better water control also exhibit traits of an avoidance strategy, including elastic tissues, high capacitance, and less negative osmotic pressure.
- These findings point to a basal, non‐stomatal mechanism of water loss control through cell membranes and/or cell walls. Potentially, this mechanism is homologous to the nonstomatal control recently identified in angiosperms, which induces unsaturated conditions in the substomatal cavities. Bryophyta presents a valuable nonstomatal model for further investigating this mechanism.

## Graphical abstract

Kinetics of cell wall water vapor potential (Ψ_cw_) and resistance of cell wall and plasmatic membrane to water flux in response to cytoplasmic water potential (Ψ_cyt_) during dehydration. Ψ_cyt_ was calculated from the WC at any moment of the dehydration curve by using the relationship between −1/Ψ_w_ and WC of pressure volume curves and assuming Ψ_w_ = Ψ_cyt_. The vertical red lines represent turgor loss point.

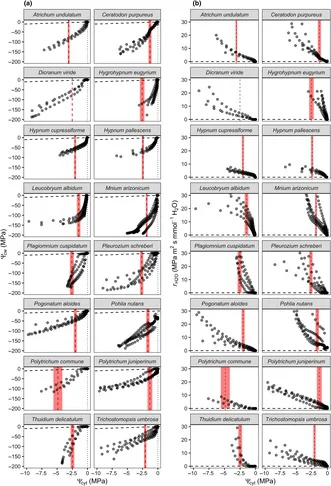

See also the Commentary on this article by Gimeno, **252**: 5–6.

## Introduction

Poikilohydry refers to plants whose water status is completely dependent on their environment (Walter, 1931), that is, inability to control water loss and isolate their cell water potential from that of the surrounding atmosphere. Poikilohydry is exhibited by lichens, gametophytes of ferns, lycophytes and bryophytes, and some sporophytes of ferns (filmy ferns, Hymenophyllaceae, Proctor, 2012). The shared characteristic of these poikilohydric groups of plants is the absence of stomata. In bryophytes (mosses, liverworts, and hornworts), stomata, when present, are limited to sporangia of sporophytes and their physiological and evolutionary constraints differ from the stomata of the leaves of tracheophytes (Renzaglia *et al*., 2020). Except for the very well‐preserved gametophytes from the Lower Devonian (Pragian) Rhynie chert (Kenrick & Crane, 1997; Edwards *et al*., 1998), no other fossils or extant gametophytes are known to have stomata (Duckett & Pressel, 2017). By contrast, homoiohydric plants maintain their water status within tight limits thanks mainly to a more or less controlled stomatal closure. This control ranges from active stomatal control in angiosperms to passive control in ferns and sporophytes of mosses and hornworts (Duckett & Pressel, 2022). Additional adaptations, such as hydrophobic cuticle, endohydry (internal effective conducting apparatus), intercellular gas space system, and structures that can take up liquid water from the soil, further enhance their ability to avoid dehydration (Jones & Dolan, 2012; Lucas *et al*., 2013; Duckett & Pressel, 2017; Xue *et al*., 2017).

Such diversity in traits of different phylogenetic groups and life phases has made several authors consider a continuum poikilohydry–homoiohydry strategy in terrestrial plants (Proctor & Tuba, 2002; Raven, 2002; Raven & Edwards, 2004; Vitt *et al*., 2014). Some bryophytes share characteristics more associated with homoiohydric plants, such as the cuticle of some liverworts (Raven, 1977, 1984, 1993), endohydry of *Polytrichum* and filmy ferns (Hébant, 1977; Ligrone *et al*., 2000; Brodribb *et al*., 2020), and intercellular air‐filled spaces of ventilated thalloid liverworts and hornworts (Duckett & Pressel, 2017). Furthermore, even when water conduction is typically external and diffuse, the growth habit of some bryophytes (gametophytes hereafter) resembles compacted shoots (small branches analogous to tracheophytes) that confer canopies the ability to retain large amounts of external capillary water (Dilks & Proctor, 1979; Proctor *et al*., 1998), at the expense of limiting CO_2_ diffusion. This also results in thick boundary layers, which allow bryophytes to lose this external water slowly without affecting cell water status (Marschall & Proctor, 2004; Rice *et al*., 2014). These characteristics, therefore, buffer the rate of water loss for a given availability of water in the environment and delay water stress (Proctor *et al*., 2007). Such ‘avoidance’ behavior of moss gametophytes has been rarely considered when discussing the poikilohydry–homoiohydry continuum (Vitt *et al*., 2014; Jabłońska *et al*., 2023). However, this short‐term storage of external capillary water is not incompatible with the ability to tolerate desiccation (up to −100 MPa; Oliver *et al*., 2020), a trait also widely observed among the studied bryophytes (Morales‐Sánchez *et al*., 2022).

In addition to retaining capillary water between the canopy structures (i.e. outside the cell walls), some bryophytes also exhibit shoot‐level internal water storage capacity, which may further contribute to delaying desiccation. Besides their high water content (WC) of the cytoplasm and capacitance of bryophytes (Proctor & Tuba, 2002; Perera‐Castro *et al*., 2020a), bryophytes present the thickest cell wall of terrestrial plants (0.6–3.5 μm, Perera‐Castro *et al*., 2022a), which contains 4%–18% of the total WC of the tissues (Proctor *et al*., 1998; Proctor, 1999; Perera‐Castro & Flexas, 2022). Cell wall thickness has been positively correlated with the recovery of carbon uptake of mosses after desiccation in a controlled dehydration treatment at 33% relative humidity (Coe *et al*., 2019). The capacity to recover functionality after desiccation has also been associated to thicker cell walls in ‘resurrection’ ferns and angiosperms (Nadal *et al*., 2021) and with higher apoplastic fraction in desiccation tolerant mosses and liverworts (Perera‐Castro & Flexas, 2022), although the underlying mechanism is still elusive. In any case, neither canopy nor shoot storage capacity operates as an active water control mechanism, since they do not affect the water transport conductance of tissues during dehydration but rather the amount of water lost before impacting the tissue water potential.

Dehydration of plant cell walls provokes physical strains of cellulose microfibrils and changes in porosity (Huang *et al*., 2018). However, among the components involved in the water pathway to the sites of evaporation (cytoplasm–cell plasma membrane–cell wall), only the plasma membrane and its structures can serve as an active regulatory barrier for controlling water loss, for example, through aquaporins (AQPs). These ubiquitous transmembrane proteins act as channels for many molecules, above all water, and have been described to be involved in a wide variety of processes, including water fluxes through plant tissues that are linked to the transpiration stream (van Dongen & Borstlap, 2004; Maurel *et al*., 2015; Kapilan *et al*., 2018; Singh *et al*., 2020; Byrt *et al*., 2023). Consistent with this observation, different expressions of AQPs resulted in different water control and drought tolerance in tracheophytes (Kapilan *et al*., 2018; Verdoucq & Maurel, 2018; Singh *et al*., 2020; Byrt *et al*., 2023). The study of AQPs in the moss *Physcomitrium patens* has revealed that this species contains the same subfamilies of AQPs that are described in angiosperms (PIP, TIP, NIP, and SIP subfamilies), as well as other exclusive subfamilies (XIP, HIP, GIP). This indicates that the main radiation of plant AQPs preceded the land plant colonization (Borstlap, 2002; Danielson & Johanson, 2008). In addition, Liénard *et al*. (2008) reported that the suppression of AQPs in *Physcomitrium* could increase its sensitivity to moderate stress conditions (visually evaluated by the shrinkage of shoots). However, the authors presented AQPs as facilitators of water entrance rather than as controllers of water loss. Since their description in mosses, AQPs have emerged as important components of the transcriptional response to water stress in this group of plants (Cuming *et al*., 2007), although researchers have been reticent to consider this as a mechanism of water control: ‘… their role in mosses has been unclear, as poikilohydric plants do not regulate their water potential’ (Charron & Quatrano, 2009).

We argue that such discoveries serve as a starting point for considering bryophyte gametophytes as poikilohydric, nonstomatal plants with a certain level of water control at the shoot level. The regulation of water flux through cell membranes by AQPs has been proposed as a nonstomatal mechanism controlling transpiration in leaves (Wong *et al*., 2022), challenging the long‐standing assumption that leaf air spaces are nearly saturated with water vapor (Cernusak *et al*., 2024; Diao *et al*., 2024). It has been reported that relative humidity in these spaces can drop to 80% under increasing vapor pressure deficit, while mesophyll cells maintain turgor (Cernusak *et al*., 2024). Even further, the intercellular humidity can drop to as low as 60% when using transgenic lines with non‐closing stomata (Cernusak *et al*., 2019). Leaves with non‐closing stomata can be considered nearly ‘cell‐wall‐naked’ to atmospheric conditions, similar to those found in poikilohydric plants. As with the lack of saturation in the leaf air space, low relative humidities on the surfaces of bryophytes – without a corresponding reduction in cytosolic water potential – would suggest a nonstomatal water control mechanism through plasma membranes and/or cell wall. If this mechanism is confirmed in mosses, it would further support the idea of water control in poikilohydric organisms and suggest a possible more ancestral origin for the nonstomatal control of transpiration.

The aim of this study was to investigate water control in the gametophytes of mosses by directly quantifying their capacity to regulate water loss through gas exchange measurements in detached moss shoots during dehydration. By quantifying water loss regulation in bryophytes, this study fills a key functional gap and provides a basis for future investigations of the underlying molecular mechanisms. We collected 16 species from Albuquerque, Boston (USA) and Tenerife (Spain) and performed gas exchange and pressure–volume measurements, along with microscopy analysis to gain further insight into moss water relations and strategies. These analyses included measurements of apoplastic and cytosolic water potential, capacitance, osmotic potential, and cell wall thickness. We hypothesized that the cell wall water potential of mosses would undergo a similar drop to that reported for angiosperms mesophyll cell walls, and likewise without a loss of turgor or equilibration with cytosolic water potential. In other words, we tested whether mosses exhibit a nonstomatal control of water loss similar to that of angiosperms.

## Materials and Methods

### Plant materials

A total of 16 species of mosses (Bryophyta phylum) were collected in the field in Sandia Crest (Albuquerque) and Billerica (Boston), both in the United States and Aguagarcía (Tenerife) in Spain (Table 1). Samples of each species were collected from similar habitats and substrates to minimize intraspecific variation. Species were selected based on their abundance in the field to ensure adequate sample material for the different measurements. Patches of 1–5 cm^2^ of moss and below‐moss soil/wood were introduced in nonsealed plastic bags and stored in growth chambers at the University of New Mexico (14 species) or at the University of Balearic Islands (2 species). The moss patches were placed together in nonhermetically sealed plastic boxes covered with a transparent plastic film. All plastic boxes were randomly distributed inside the growing chamber. Mosses were maintained irradiated with 430–460 μmol m^−2^ s^−1^ of light with a photoperiod of 14 h : 10 h and a moss temperature of 21–23°C during the day and 16–20°C during the night. Moss temperatures were measured by placing a thermocouple on the canopy surface of the mosses (EL‐USB‐TC; Lascar Electronics Inc., Dayton, OH, USA). The surfaces of the mosses were sprayed with distilled water once a week. All measurements were performed 1–3 months after collection. Mosses were separated from their substrate and washed with distilled water for removing soil or wood particles before the measurements. Excess external water was also removed by gently pressing each sample against a piece of filter paper. This sample manipulation did not cause dehydration, as gas‐exchange and water‐potential analyses were started on samples that still contained more water than the fully hydrated state (RWC > 100%). In all cases, samples consisted of several clean shoots from one or several patches without brown nonphotosynthetic tissues. Species were identified according to Casas *et al*. (2006); Atherton *et al*. (2010); Pope (2016); Allred *et al*. (2024).

**Table 1:** List of studied species; identification and general ecology information are based on Atherton *et al.* (2010); Casas *et al.* (2006); Pope (2016); Allred *et al.* (2024).

Species	Family	Location	Growth habit	Habitat/ecology
*Atrichum undulatum* (Hedw.) P. Beauv.	Polytrichaceae	Billerica, MA	Acrocarpous	In shaded, well‐drained places, only avoiding most acidic and highly calcareous soils
*Ceratodon purpureus* (Hedw.) Brid.	Ditrichaceae	Sandia Crest, NM	Acrocarpous	Wet to dry exposed soil, disturbed, acidic, well‐drained sites
*Dicranum viride* (Sull. & Lesq.) Lindb.	Dicranaceae	Billerica, MA	Acrocarpous	On shaded and humid places, moderate acidic bark‐dwelling
*Hygrohypnum eugyrium* (Schimp.) Loeske	Amblystegiaceae	Billerica, MA	Pleurocarpous	On rocks and stones in swift‐flowing streams in upland regions
*Hypnum cupressiforme* Hedw.	Hypnaceae	Sandia Crest, NM	Pleurocarpous	On acidic to slightly base‐rich bark and siliceous rock
*Hypnum pallescens* (Hedw.) P.Beauv.	Hypnaceae	Billerica, MA	Pleurocarpous	Rotten wood and tree bases
*Leucobryum albidum* (Brid. ex P.Beauv.) Lindb.	Leucobryaceae	Billerica, MA	Acrocarpous	Well‐drained, rock outcrops
*Mnium arizonicum* Amann	Mniaceae	Sandia Crest, NM	Acrocarpous	Dry to moist soil and humus in the mountains
*Plagiomnium cuspidatum* (Hedw.) T. Kop.	Mniaceae	Billerica, MA	Acrocarpous	On wet to damp soil, humus, rotting logs, and rocks
*Pleurozium schreberi* (Brid.) Mitt.	Hylocomiaceae	Billerica, MA	Pleurocarpous	On forest floors in the mountains, avoiding calcareous or base‐rich habitats
*Pogonatum aloides* (Hedw.) P.Beauv.	Polytrichaceae	Aguagarcía, Tenerife	Acrocarpous	On damp, shady, acidic slopes
*Pohlia nutans* (Hedw.) Lindb	Mniaceae	Sandia Crest, NM	Acrocarpous	On wet or dry, acidic soil, also bogs and tree bases
*Polytrichum commune* Hedw.	Polytrichaceae	Billerica, MA	Acrocarpous	Acidic moist organic soils, meadows, fens
*Polytrichum juniperinum* Hedw.	Polytrichaceae	Aguagarcía, Tenerife	Acrocarpous	On exposed, acidic soils and slopes, from the lowlands to the high mountains
*Thuidium delicatulum* (Hedw.) Schimp.	Thuidiaceae	Billerica, MA	Pleurocarpous	On shaded moist rock and soil banks in the mountains
*Trichostomopsis umbrosa* (Müll.Hal.) H.Rob.	Pottiaceae	Sandia Crest, NM	Acrocarpous	Moist, shaded, calcareous places

Acrocarpic: compact growth with capsules at the tip of the stems. Pleurocarpic: spreading growth with capsules along the sides of the stems.

### Gas exchange measurements

Measurements of net CO_2_ assimilation rate (*A*_N_) and transpiration rate (*E*) in response to variations in WC (‘dehydration curve’) were done with an LI‐6800 gas exchange system (LiCOR Biosciences, Lincoln, NE, USA). Samples were watered to excess with distilled water before the measurements. They were then placed over a custom‐made cuvette consisting of a 6 cm^2^ gasket fixed to a piece of thin polyester stocking fabric as detailed in Perera‐Castro *et al*. (2020b) (Supporting Information Fig. S1). The cuvette containing the sample was placed inside the LI‐6800 chamber, ensuring that the cuvette and chamber gaskets were aligned. The measured projected, nonoverlapping shoot area was between 2.4 and 3.7 cm^2^. Additionally, measurements were performed on excised, nonoverlapping leaves for *Pogonatum aloides* and *Polytrichum juniperinum*. The water concentration of the incoming air (reference water) was set to 10 mmol mol^−1^ air. The flow rate within the chamber was maintained at 400 μmol s^−1^ and the CO_2_ concentration in the incoming air was set at 420 μmol mol^−1^ air. Irradiance was set at a saturation level of 800 μmol m^−2^ s^−1^ standardized for all species (tested previously with fast light‐response curves). The air temperature inside the chamber was maintained between 25.9°C and 27.6°C at the beginning of the measurements, experiencing a heating of 0–0.98°C during the dehydration curve. Moss temperature was determined by using energy balance (default calculations of LI‐6800). Gas exchange recordings were alternated with weighing of the sample each 2–5 min to obtain the fresh weight (FW) during the dehydration curve. Water content at any given time during dehydration was calculated as follows: WC = (FW − DW)/DW, where DW is the dry weight obtained at the end of the experiment by keeping the samples at 70°C for 48 h. An entire cycle of desiccation lasted for 20–45 min and stopped when *A*_N_ was close to zero. Independent samples were used for measuring dark respiration (*R*_D_) by introducing the sample and the custom‐made cuvette in the LI‐6800 chamber under dark conditions for 5 min. Due to low gas exchange rates, measurements of CO_2_ leakage (Flexas *et al*., 2007) were done regularly with an empty cuvette at the beginning and end of each dehydration curve. *A*_N_ and *E* at optimum WC (WC_opt_, that is at maximum *A*_N_) were termed *A*_opt_ and *E*_opt_, respectively. Maximum *E* (*E*_max_) along the entire dehydration curve could be observed at optimum WC or higher. *A*_N_, *R*_D_, and *E* were expressed per projected area in both acrocarpic and pleurocarpic species, obtained by an image processing program (ImageJ, NIH, Rockville, MD, USA). Independent samples (*n* = 3–4) were used to quantify sample shrinkage during dehydration (Fig. S2), ensuring that gas exchange rates are consistently expressed based on the actual projected area (Methods S1). *E* was also expressed by total leaf area. Shoot mass area (SMA) was calculated as the ratio of DW and the projected shoot area. For each species, a total of 3–5 dehydration curves were performed.

### Pressure–volume curves

Between 4 and 6 pressure–volume curves per species were performed (except for *Dicranium* sp., for which *n* = 1 due to the scarcity of plant material) by slowly air‐drying fully hydrated samples. Measurements of weight and water potential (Ψ_w_) were alternated during dehydration. Ψ_w_ was determined by using a psychrometer (model WP4C, Decagon Device Inc.). The measured ground, nonoverlapping area that was introduced in the WP4C cuvette was between 2.8 and 7.2 cm^2^. The turgid weight (TW) of each specimen was estimated as the x‐intercept of Ψ_w_ vs FW at any time on the curve (FW), avoiding the ‘plateau effect’ of extracellular water (Proctor *et al*., 1998; Hájek & Beckett, 2008). Both WC and relative water content (RWC) were calculated, the latter as RWC = 100(FW − DW)/(TW − DW). The saturating water content (SWC) was calculated as SWC = (TW − DW)/DW and SWC_area_ = (TW − DW)/projected shoot area. As for gas exchange samples, the projected shoot area was obtained with ImageJ and the SMA was also calculated as the ratio of DW and the projected shoot area. The point from which the pressure–volume curve (−1/Ψ_w_ vs RWC) became linear was considered the turgor loss point (TLP). This TLP was determined considering the *R* ^2^ of the −1/Ψ_w_ vs RWC relationship. RWC, WC, and Ψ_w_ measured at that point were obtained (RWC_tlp_, WC_tlp_, and π_tlp_, respectively). For WC higher than WC_tlp_, Ψ_w_ reflects the combined contributions of the pressure potential (Ψ_P_), arising from the hydrostatic pressure exerted by the cell contents against the cell wall, and the osmotic component Ψ_o_, arising from solutes' chemical pressure. By definition, for WC below WC_tlp_, Ψ_P_ equals 0 and Ψ_w_ is determined solely by the osmotic component, such that Ψ_w_ = Ψ_o_. The osmotic contribution can be expressed in pressure units as the osmotic pressure (π). Osmotic pressure at full rehydration (π_o_) was calculated as the inverse of the y‐intercept of the linear portion of the pressure–volume curve, below the turgor loss point. The bulk modulus of elasticity (ε) was calculated as the slope of pressure potential vs total RWC. Absolute capacitance at full turgor (*C*_ft_) and at turgor loss point (*C*_tlp_) was determined from the relationship between RWC and Ψ_w_ above and below turgor loss point, respectively. The extracellular apoplastic fraction (*a*_f_) was considered the fraction of RWC when osmotic pressure approaches −∞ and was calculated as the x‐intercept of the pressure‐volume curve. The equivalent WC that corresponds to *a*_f_ (WC_apo_) was also obtained for the −1/Ψ_w_ – WC relationship. Cytosolic (or symplastic) fraction (*c*_f_) and cytosolic water content (WC_cyt_) were also obtained as *c*_f_ = 1−*a*_f_ and WC_cyt_ = SWC−WC_af_. A graphic illustration of the pressure–volume‐derived parameters and their calculation is represented in Fig. S3.

In addition to those volumetry measurements, additional independent samples were selected for exploring other anatomical cell parameters: cell area (*A*_cell_), cell wall thickness (*T*_cw_), and the total leaf area per shoot area (*A*_leaf_/*A*_shoot_) (Methods S2).

### Calculation of cell wall water potential

At any moment of the dehydration curve, the water vapor concentration over the cell wall (*W*_cw_) was calculated from the Fick's law of diffusion equation from a nonstomatal interrupted cell wall through the boundary layer to the mixed atmosphere:

(Eqn 1) $$W_{\mathrm{cw}}=\frac{E}{g_{\mathrm{bw}}}+W_{a}$$

where *g*_bw_ is the boundary layer conductance for water and *W*_a_ is the water vapor concentration surrounding the plants (air exiting the chamber). Only *E* based on projected shoot area was used in Eqn 1. *g*_bw_ was obtained from LI‐6800 empirical calculations for a broad surface, assuming *g*_bw_ is the same on both sides of the surface. Then, the saturated water vapor pressure over the cell wall (*W*_cw,sat_) was calculated as a function of shoot temperature (Buck, 1981):

(Eqn 2) $$W_{\mathrm{cw},\mathrm{sat}}=0.6135e^{\left(\frac{17.502T}{240.97+T}\right)}$$

where *T* is the temperature of the shoot, estimated from energy balance calculations of the LI‐6800. Note that *T* estimations by energy balance require the use of net radiation, which is based on projected shoot area, and latent heat flux, which is calculated from *E*. Therefore, this calculation can only be done using *E* based on projected area of the sample (shoots or leaves). The relative humidity at the cell wall surface (RH_cw_) was then calculated as follows:

(Eqn 3) $${\mathrm{RH}}_{\mathrm{cw}}\left(\%\right)=\frac{W_{\mathrm{cw}}}{W_{\mathrm{cw},\mathrm{sat}}}100$$

The RH_cw_ measured at optimum WC (WC_opt_, that is at maximum *A*_N_) was termed RH_cw,opt_. The shoot surface cannot be considered completely flat, impacting *g*_bw_ and affecting temperature estimates by energy balance, which also depend on *g*_bw_. Since this effect is unavoidable, a sensitivity analysis was performed to determine the variability of RH_cw,opt_ with changes in *g*_bw_ (Fig. S4) and *T* (Fig. S5). An absolute variation of *g*_bw_ in 1.6 mol m^−2^ s^−1^ did not provoke significant variation in RH_cw,opt_ directly or by changes in energy balance *T* (Fig. S4). An averaged change in RH_cw,opt_ of 5.1% ± 0.8% for 1°C of error in estimates of *T* was calculated for all species (Fig. S5). The sensitivity of energy balance *T* to the moss absorbance (α) was also estimated (Fig. S6), resulting in a maximum change in *T* of 0.5°C for a variation of α between 0.6 and 1.

Water vapor potential at the cell wall (Ψ_cw_) was calculated by the Kelvin equation as described in Nobel (2020):

(Eqn 4) $$\Psi_{\mathrm{cw}}=\frac{\mathrm{RT}}{M_{w}}\ln\left(\frac{W_{\mathrm{cw}}}{W_{\mathrm{cw},\mathrm{sat}}}\right)+p_{w}\mathrm{gh}$$

where *R* is the universal gas constant (8.314 J mol^−1^ K^−1^), *T* is the temperature, *M*_w_ is the partial molar volume of water (18 × 10^−6^ m^3^ mol^−1^), *p*_w_ is water density, *g* is the gravitational acceleration, and *h* is altitude (1508 m above sea level). The equation was conveniently rescaled to MPa (*RT*/*M*_w_ = 137.3 MPa).

Since most of the water of a nonfully hydrated shoot is located within the living cells, this likely dominates the determination of whole‐tissue water potential, as assumed for leaves (Wong *et al*., 2022; Scoffoni *et al*., 2023). Therefore, cytosolic water potential (Ψ_cyt_) was assumed to be equal to Ψ_w_ estimated at any moment of the dehydration curve from the −1/Ψ_w_ – WC relationship of the pressure‐volume curves (polynomial or exponential curve fitting, Fig. S7). The resistance of cell wall/plasmatic membrane to water movement (*r*_H2O_) was calculated based on a linear relation between water flux and the water potential gradient between cytosolic and cell wall:

(Eqn 5) $$r_{H2O}=\frac{\Psi_{\mathrm{cyt}}-\Psi_{\mathrm{cw}}}{E}$$

The value of *r*_H2O_ at the moment when the cell wall loses turgor was estimated using the mean π_tlp_ of each species.

### Statistical analysis

All analyses were performed using the R statistical software (R Core Team, 2023). Some special packages were used: plyr (Wickham, 2011), ggplot2 (Wickham, 2016). Intraspecific differences in transpiration rate at different moments of dehydration were evaluated by a paired *t*‐test. Differences in the transpiration rates of the studied species were evaluated by the Kruskal–Wallis test. To discern the biological/methodological origin of such differences in transpiration, linear regression between transpiration and related chamber conditions (vapor pressure and temperature) was performed. Linear regression was also used for testing the relationship between the calculated RH_cw,opt_ and possible determinants, like boundary layer conductance involved in its calculation, or morphological parameters theoretically involved with the amount of water available in the sample for its exchange, such as SWC per shoot area, or with the exchange area of samples, such as *A*_leaf_/*A*_shoot_ (Methods S2). The relationship between RH_cw,opt_ and parameters derived from the pressure‐volume was also explored. All parameters used in this manuscript are summarized in Table 2.

**Table 2:** Summary of the parameters used in this study.

Acronym	Units	Definition
*A*_cell_	μm^2^	Averaged surface area of cells
*a*_f_	–	Apoplastic water fraction
*A*_leaf_/*A*_shoot_	–	Total leaf (phyllids) area per shoot area
*A*_N_	μmol CO_2_ m^−2^ s^−1^	Net CO_2_ assimilation rate
*A*_opt_	μmol CO_2_ m^−2^ s^−1^	Maximum net CO_2_ assimilation rate of the dehydration curve
*c*_f_	–	Cytosolic (symplastic) water fraction
*C*_ft_	MPa^−1^	Capacitance at full turgor, that is, the slope between water potential and relative water content above turgor loss point
*C*_tlp_	MPa^−1^	Capacitance at turgor loss point, that is, the slope between water potential and relative water content below turgor loss point
*E*	mmol H_2_O m^−2^ s^−1^	Transpiration rate
*E*_max_	mmol H_2_O m^−2^ s^−1^	Maximum transpiration rate of the dehydration curve
*E*_opt_	mmol H_2_O m^−2^ s^−1^	Transpiration rate at maximum *A*_N_
*g*_bw_	mol H_2_O m^−2^ s^−1^	Boundary layer conductance for water
*R*_D_	μmol CO_2_ m^−2^ s^−1^	Dark respiration rate
*RH*_cw_	%	Relative humidity at the cell wall surface
*RH*_cw,opt_	%	Relative humidity at the cell wall surface when *A*_N_ is maximum (at WC_opt_)
RWC	%	Relative water content. Percentage of water relative to maximum amount of water
RWC_tlp_	%	Relative water content at turgor loss point (when pressure potential became cero)
*r*_H2O_	MPa m^2^ s mmol H_2_O	Resistance of cell wall/plasmatic membrane to the flux of H_2_O
MA	g m^−2^	Shoot mass area
SWC	g H_2_O g^−1^ DW	Saturated water content of mosses tissues per dry mass (assumed to be equal to WC_opt_, at maximum *A*_N_)
SWC_area_	g H_2_O m^−2^	Saturated water content of mosses tissues per ground area
*T*_cw_	μm	Cell wall thickness
WC	g H_2_O g^−1^ DW	Water content of mosses tissues, it is, amount of water per unit dry weight at any time of the dehydration curve
WC_apo_	g H_2_O g^−1^ DW	Apoplastic water content, that is, the amount of saturating apoplastic water per unit dry weight
WC_cyt_	g H_2_O g^−1^ DW	Cytosolic water content, that is, the amount of saturating cytosolic water per unit dry weight
WC_opt_	g H_2_O g^−1^ DW	Water content of mosses tissues at maximum *A*_N_
WC_tlp_	g H_2_O g^−1^ DW	Water content of moss tissues at turgor loss point, when pressure potential is cero
*W*_cw_	MPa	Water vapor concentration over the cell wall
*W*_cw,sat_	MPa	Saturated water vapor pressure over the cell wall
Ψ_w_	MPa	Water potential is thermodynamic term that quantifies the free energy of water and therefore determines the direction of water movement
Ψ_o_	MPa	Osmotic potential, suction provoked by the concentration of dissolved solutes
Ψ_P_	MPa	Pressure (turgor) potential, the physical pressure exerted by the cell contents against the cell wall
ε	MPa	Bulk modulus of elasticity, that is, the slope of pressure potential vs relative water content
π_o_	MPa	Osmotic pressure at full hydration
π_tlp_	MPa	Osmotic pressure at turgor loss point
*Ψ*_cw_	MPa	Water vapor potential at the cell wall
Ψ_w_ = Ψ_cyt_	MPa	Water potential of mosses tissues, assumed to be equivalent to cytosolic water potential

## Results

### Mosses present different transpiration rates and dehydration kinetics

**Fig. 1:**
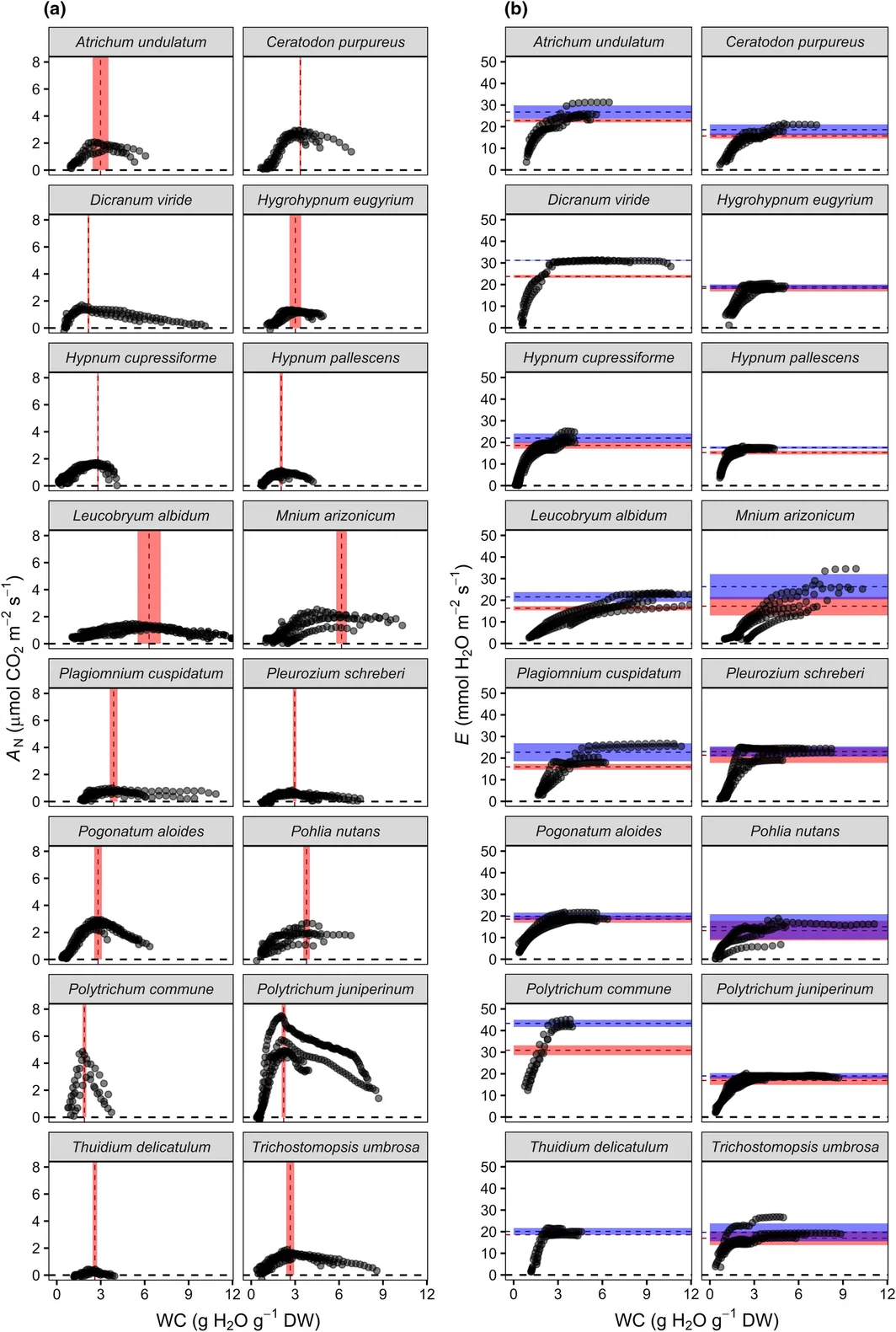
Response to dehydration of (a) net CO_2_ assimilation rate (*A*_N_) and (b) transpiration rate (*E*) for the studied species. Red vertical lines indicate the average water content (WC) at which maximum *A*_N_ (*A*_opt_) is found (WC_opt_). Red and blue horizontal lines indicate averaged transpiration rates at WC_opt_ (*E*_opt_) and maximum transpiration rates (*E*_max_), respectively. Shaded areas indicate ±SD of the respective parameters.

**Fig. 2:**
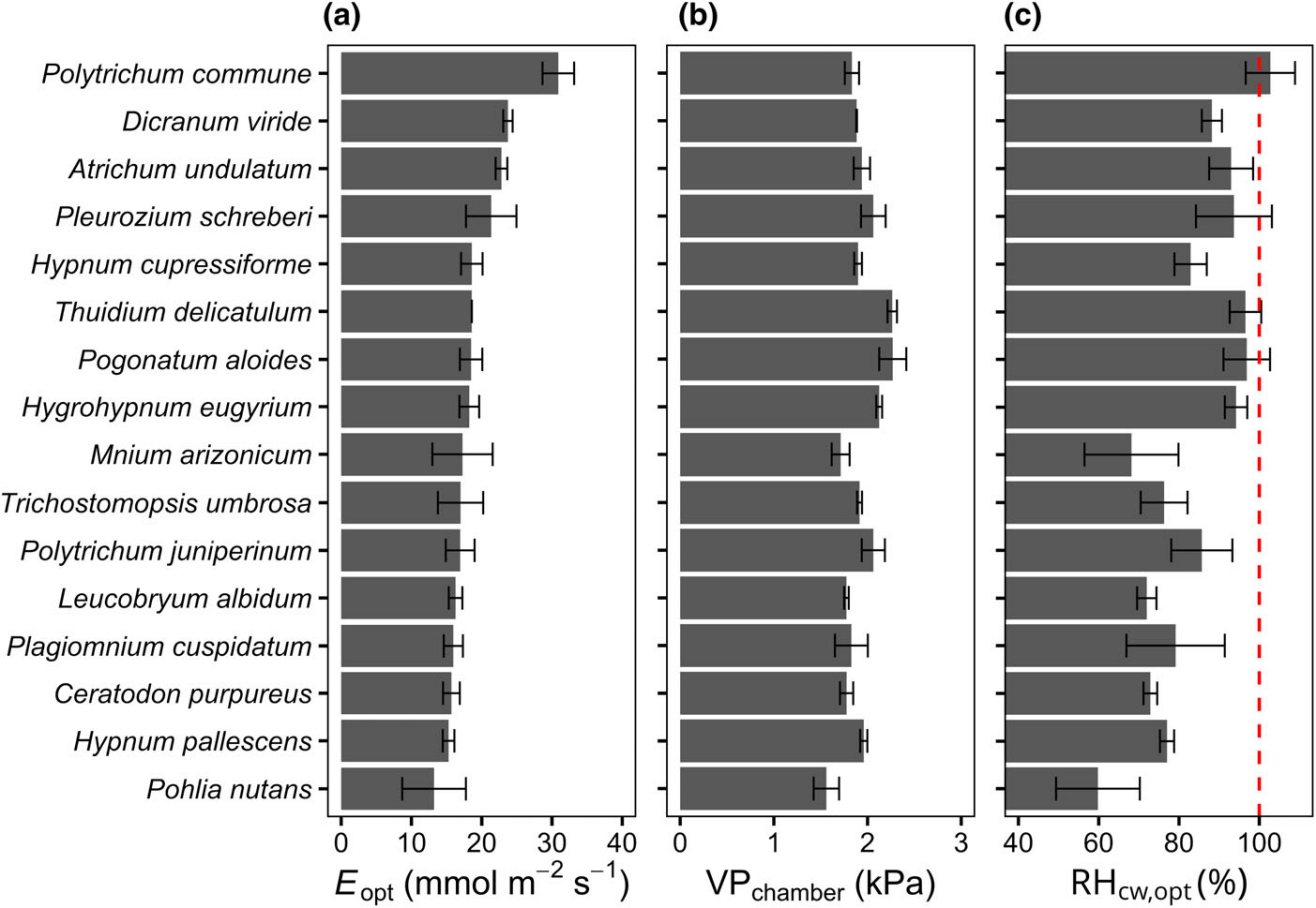
Interspecific variation of the (a) transpiration rate at the maximum net CO_2_ assimilation (*E*_opt_), (b) vapor pressure inside the chamber (VP_chamber_) at an air temperature of 26.6 ± 0.5°C and an incoming H_2_O concentration of 9.98 ± 0.03 mmol mol^−1^, and (c) relative humidity at the cell wall in optimum water content (RH_cw,opt_). The maximum difference in sample and reference water vapor concentrations varied between 4 and 17 mmol mol^−1^. *E*_opt_, VP_chamber_ and RH_cw,opt_ significantly differ between species (*P* < 0.001, Kruskal–Wallis test), although *E*_opt_ and RH_cw,opt_ were not associated with VP_chamber_ (nonsignificant linear regression). Bars indicate mean ± SD. Dashed red line in (c) indicates maximum theoretical value of RH_cw,opt_ (100 %).

*A*_N_ responses to dehydration described a parabolic curve in all studied species, with a decrease in *A*_N_ for any WC higher or lower than WC_opt_ (Fig. 1a). WC_opt_ varied between 1.9 ± 0.1 g H_2_O g^−1^ DW in *Polytrichum commune* to 6.3 ± 0.8 g H_2_O g^−1^ in *Leucobryum albidum* (mean ± SD). By contrast, the transpiration rate remained at its maximum value (*E*_max_) for any WC higher than WC_opt_ (Fig. 1b). This asymptotic value of *E*_max_ was significantly higher than *E*_opt_, except in *Pohlia nutans* and *Thuidium delicatum* (paired *t*‐test, *P* > 0.001), indicating that in most studied species, the transpiration rate started decreasing before maximum assimilation rate during dehydration (at WC > WC_opt_). This was also observed in detached leaves of *P. aloides* and *P. juniperinum* (Fig. S8). Significant interspecific differences were observed in both *E*_opt_ (Fig. 2a) and *E*_max_, ranging from a *E*_opt_ of 30.9 ± 2.2 μmol m^−2^ s^−1^ in *P. commune* (*E*_max_ = 43.3 ± 1.6 μmol m^−2^ s^−1^) to a *E*_opt_ of 13.2 ± 4.5 μmol m^−2^ s^−1^ (*E*_max_ = 14.9 ± 5.8 μmol m^−2^ s^−1^) in *P. nutans*. The studied species also differ in their *E*_opt_ expressed by total exposed area (3.5–21.3 mmol m^−2^ s^−1^) (Table S1). Such differences were not correlated with *A*_opt_ (data not shown) or with the vapor pressure inside the chamber (Fig. 2b), which resulted from an air temperature interspecific variation of 26.6 ± 0.5°C and an incoming H_2_O concentration of 9.98 ± 0.03 mmol mol^−1^. From projected area‐based *E*_opt_, the calculated values of RH_cw,opt_ showed also significant differences between species, ranging from values not significantly different from saturation (102.7% ± 6.1%) to 59.8% ± 10.4% in *P. commune* and *P. nutans*, respectively (Fig. 2c). Detached leaves also showed low RH_cw,opt_ values (74% on average) (Fig. S9). This range of RH_cw,opt_ corresponded to values of Ψ_cw_ from 0 to −58.9 MPa (Fig. S10). RH_cw,opt_ was mainly correlated with *E*_opt_, from which is calculated, and not with *g*_bw_ or any anatomical parameter examined here, including those related with the amount of water that samples can hold and provide per unit area (SWC_area_, both in the apoplastic and the symplastic) or with the exchange surface, such as the *A*_leaf_ : *A*_shoot_ ratio (Fig. S11). The time required for dehydration (from *A*_opt_ to *A*_N_ = 0), which took from 8 to 31 min, was not significantly correlated with *A*_opt_, *E*_opt_, or *E*_max_.

### Moss cell walls can sustain lower water potential without cytosolic dehydration

**Fig. 3:**
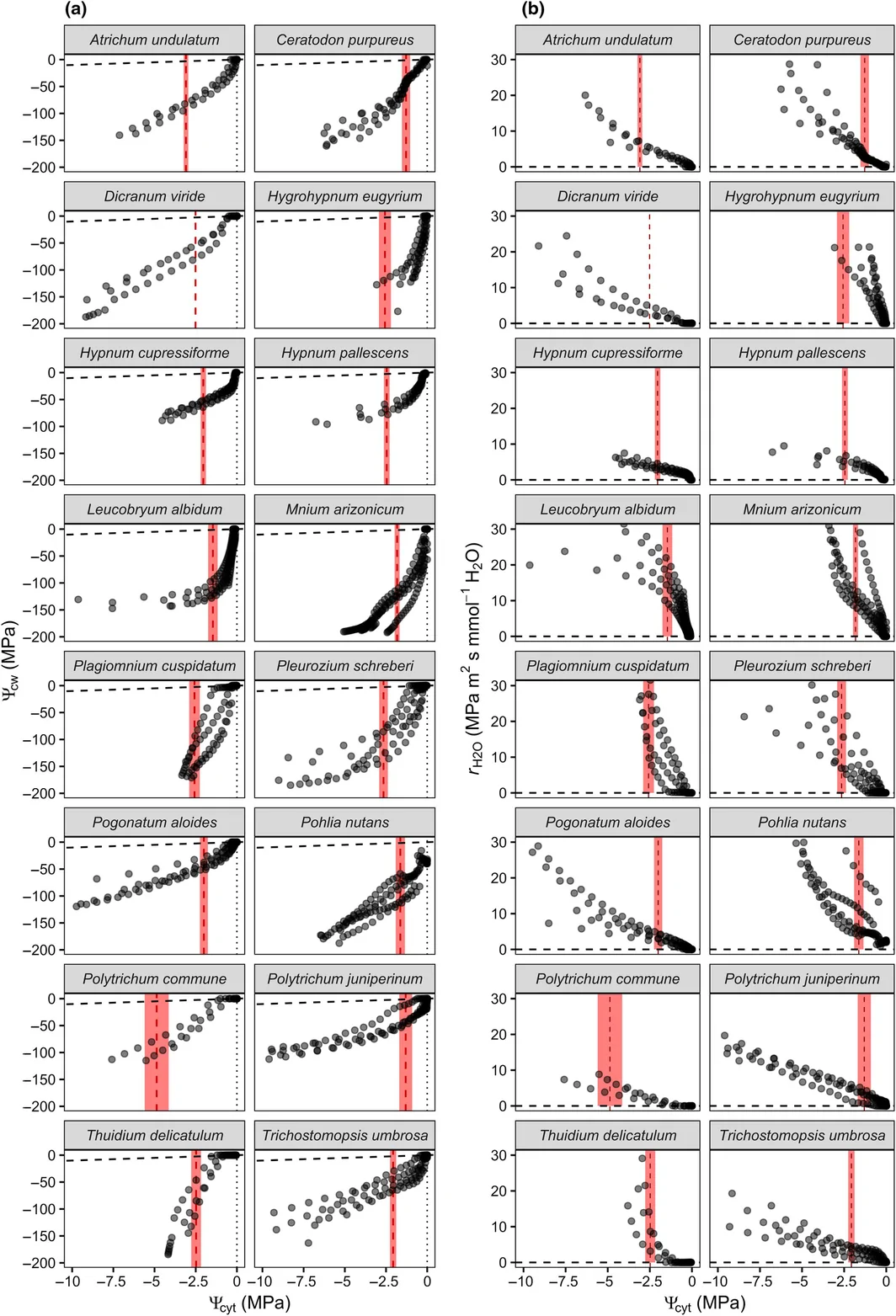
Kinetics of (a) cell wall water vapor potential (Ψ_cw_) and (b) resistance of cell wall and plasmatic membrane to water flux in response to cytoplasmic water potential (Ψ_cyt_) during dehydration. Ψ_cyt_ was calculated from the WC at any moment of the dehydration curve by using the relationship between −1/Ψ_w_ and WC of pressure volume curves (Supporting Information Fig. S7) and assuming Ψ_w_ = Ψ_cyt_. The vertical dashed red line represents the averaged water potential at the turgor loss point (π_tlp_) ± SD. The diagonal dashed black line indicates a 1 : 1 relationship.

For WC < WC_opt_, Ψ_cw_ was significantly lower than Ψ_cyt_ in all studied species (Fig. 3a), both in shoots and detached leaves (Fig. S12). When *A*_N_ was still positive in most of the species and Ψ_cyt_ reached π_tlp_ values, Ψ_cw_ could be 10 orders of magnitude more negative than Ψ_cyt_. This is consistent with high resistances to water fluxes in all studied species at the turgor loss point (Fig. 3b), with significant interspecific differences (*r*_H2O_ between 2.79 ± 0.45 MPa m^2^ s mmol^−1^ H_2_O for *Ceratodon purpureus* and 23.6 ± 14.36 MPa m^2^ s mmol^−1^ H_2_O for *Plagiomnium cuspidatum*). Four out of five dehydration curves of *Hygrohypnum eugyrium* reached *A*_N_ = 0 (and the dehydration curve was interrupted) before reaching the turgor loss point. For all other species, *A*_N_ was still positive when the plants crossed π_tlp_, indicating these plants are still active at low cell wall water potential.

### Cell wall surface humidity correlated with dehydration avoidance traits

**Fig. 4:**
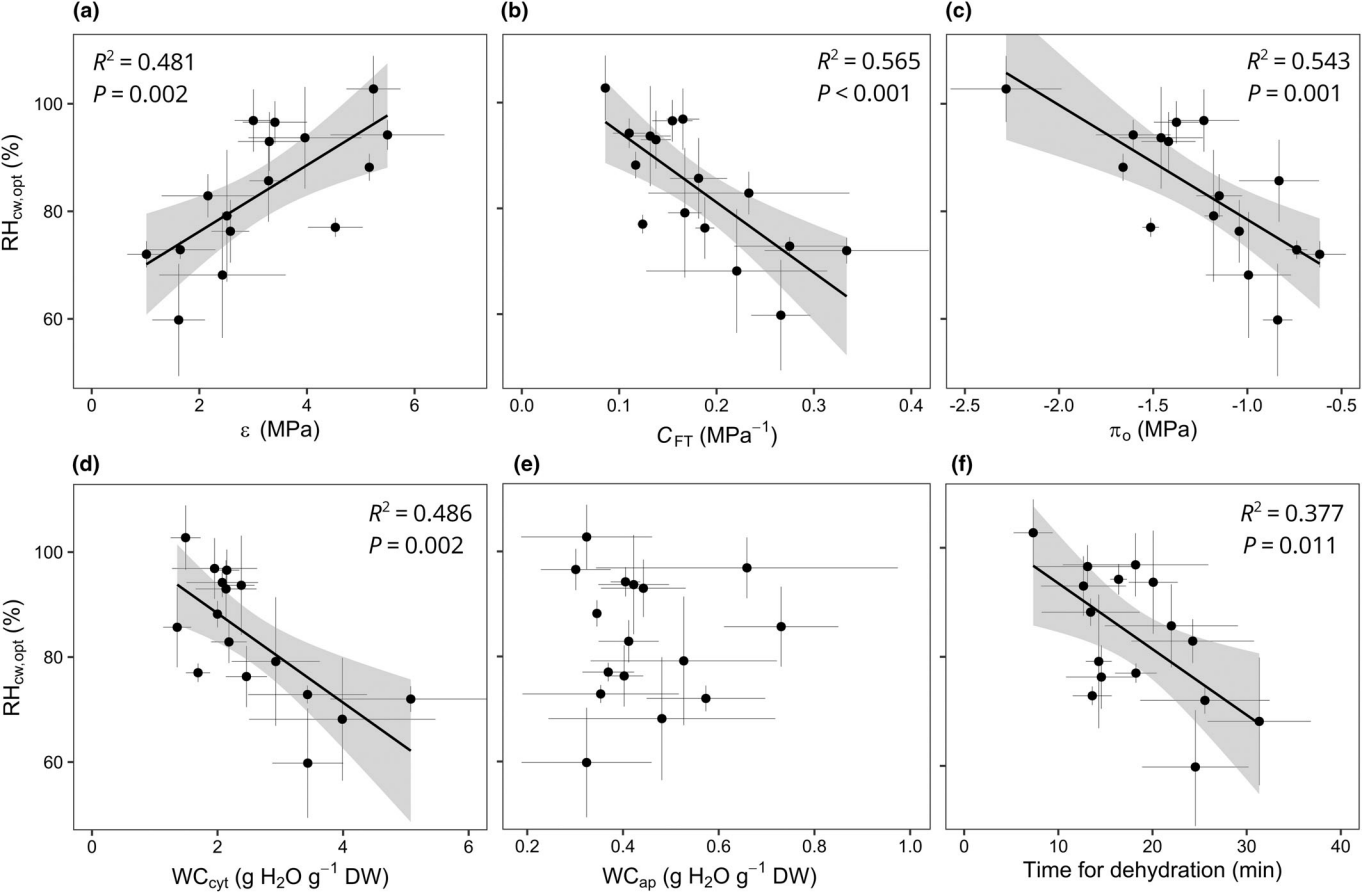
Relationship between relative humidity at the cell wall in optimum water content (RH_cw,opt_) and water‐related traits: (a) bulk modulus of elasticity (ɛ), (b) capacitance for water content range above turgor loss point (*C*_ft_), (c) osmotic potential (π_o_), (d) cytosolic water content per unit dry weight (WC_cyt_), (e) apoplastic water content per unit dry weight (WC_ap_), and (f) time for dehydration (from *A*_opt_ to *A*_N_ = 0 in the dehydration curves). Points represent the mean ± SD for each species (*n* = 3–5 for RH_cw_ and time for dehydration, *n* = 1–6 for pressure‐volume derived parameters).

Some pressure–volume‐derived parameters, which were measured in independent samples of the same species, showed a significant correlation with the relative humidity calculated for cell walls (Fig. 4a–c). Highest RH_cw,opt_ values were measured in species with the highest bulk modulus of elasticity (higher ɛ, more rigidity, Fig. 4a), with the lowest capacitance (*C*_FT_, Fig. 4b) and more negative osmotic pressure (π_o_, Fig. 4c) at full turgor. The species with the highest CWC per unit dry weight (WC_cyt_) presented with the lowest RH_cw,opt_ (Fig. 4d), independently of the apoplastic WC (Fig. 4e). These profiles of traits (more elastic tissues, higher capacitance, low osmotic adjustment, and high water storage) corresponded to the species whose dehydration curves took longer to finalize (from *A*_opt_ to *A*_N_ = 0): *Mnium arizonicum*, *L. albidum*, and *P. nutans* (Fig. 4f).

## Discussion

### Evidence for control of water loss in mosses

As introduced, poikilohydry has been conceived as an inability to control water loss (Proctor & Tuba, 2002; Raven, 2002; Raven & Edwards, 2004; Vitt *et al*., 2014), equivalent to a nearly infinite conductance of tissues to water only driven by capillary forces and boundary layer conductance. In fact, the reported shoot maximum rates of transpiration for the studied mosses (15–30 mmol m^−2^ s^−1^) are more than 10‐fold higher than those reported for homoiohydric plants (below 4 mmol m^−2^ s^−1^ for most reports, Kattge *et al*., 2020, TRY database, mostly angiosperms), whose gas exchange capacities are limited mainly by stomatal density and stomatal pore size under nonwater–stress conditions (Fanourakis *et al*., 2015; Franks *et al*., 2015). However, the result of this study provides evidence supporting partial control of water loss in mosses. At the moment when WC_opt_ was reached, humidity values at the surface of the cell wall (RH_cw,opt_) calculated from *E*_opt_ were lower than 100% in most species, going as low as 60% in *P. nutans* (i.e. not reflecting equilibrium with cytosolic water). The range of RH_cw,opt_ values reported in our study (60–100%) for mosses at WC_opt_ are close to those measured for angiosperms internal airspaces (60–93%, Cernusak *et al*., 2024), including those studies where stomatal opening is induced somehow (60–70%, Jarvis & Slatyer, 1970; Cernusak *et al*., 2019). Those ranges of RH_cw,opt_ and the discrepancies between Ψ_cw_ and Ψ_cyt_ (*r*_H2O_ >> 0) point to partially regulated water loss and argue against the view that water loss in mosses is entirely uncontrolled.

Although transpiration rates were high, they differed between species even under similar chamber vapor pressure and incoming air dryness, regardless of whether *E* was calculated based on projected area or total exposed area. Interspecific differences in transpiration and rates of water loss have been previously reported in mosses (Krupa, 1977; Goetz & Price, 2015). Even evapotranspiration of moss‐covered soils has been described to be significantly lower than from bare soils, suggesting that the moss layer acts as a barrier to water vapor transfer between the soil and the atmosphere (Liu *et al*., 2022). Authors have normally explained these results by the physical properties of the moss layer, such as capillary water storage and canopy structure, rather than by a physiological control of water loss. To our knowledge, only Krupa (1977) considered an interspecific different resistance to evaporation maybe related to different properties or structure of cell walls. Taken together, these studies attribute interspecific differences primarily to structural properties of the moss canopy rather than to intrinsic physiological control, providing a framework against which our results can be interpreted.

Our results were obtained for detached and non‐overlapping shoots that do not conserve their canopy water retention properties. The compulsory presence of the stem (caulidium) during the measurement of the sample must contribute to the water flux, as demonstrated in two Polytrichaceae species where entire shoots presented higher values of *E*_opt_ than detached leaves (Fig. S9). Thus, any evidence of water control reported in our study must be the result of an averaged behavior of a heterogeneous sample, with different proportions of leaves and caulidia, whose tissues would likely present different levels of water control. The percentage of caulidia in projected shoot area or shoot weight (reported in Table S1 for some species) did not explain the variations in *E*_opt_ and derived parameters obtained in our study. The same happened for the ratio of leaf areas per projected shoot area (*A*_leaf_/*A*_shoot_). The lack of correlation between *E*_max_ or *E*_opt_ and *A*_leaf_/*A*_shoot_ suggests that factors beyond shoot structure contribute to the observed variation.

As expected, dehydration of mosses provoked a decrease in transpiration rates, as already reported by Williams & Flanagan (1996). Together with transpiration, RH_cw_ and the equivalent water potential (Ψ_cw_) also decrease during desiccation. However, at the turgor loss point, Ψ_cw_ was clearly more negative than the cytosolic water potential (Ψ_cyt_) estimated from pressure to volume curves for the corresponding WC. A decoupled evolution of Ψ_cw_ and Ψ_cyt_ requires a finite hydraulic conductance across the plasma membrane (*r*_H2O_ between 2.7 and 23.6 MPa m^2^ s mmol^−1^ H_2_O), which is consistent with principles of dynamic flow control. In tracheophytes, hydraulic resistances of hydrated leaves (typically denoted 1/*K*_leaf_) range between 0.1 and 0.6 MPa m^2^ s mmol^−1^ H_2_O (Brodribb *et al*., 2005; Sack & Holbrook, 2006; Scoffoni *et al*., 2011; Hernandez‐Santana *et al*., 2016), two orders of magnitude lower than the *r*_H2O_ reported for our mosses at turgor loss point. Even angiosperms cavitated until 80% loss of hydraulic conductivity (P_80_) presented lower leaf hydraulic resistance than our studied mosses at turgor loss point (Scoffoni *et al*., 2011). Moss species with an RH_cw,opt_ close to 100% under optimum hydric conditions (*P. commune*, *P. aloides*, and *Thuidium delicatulum*) also showed significant differences between Ψ_cw_ and Ψ_cyt_ under desiccation (*r*_H2O_ between 1.74 and 2.05 MPa m^2^ s mmol^−1^ H_2_O). This suggests a nonconstitutive control of water loss in these species, with resistance becoming more pronounced under dehydration.

During dehydration of excessively hydrated cells, transpiration rates started decreasing before registering the maximum assimilation rate (*A*_opt_), that is, when WC was still higher than WC_opt_. This was evidenced by the general significant differences between *E*_max_ and *E*_opt_ (Fig. 1). Typically, *A*_opt_ of bryophytes is considered close to 100% RWC, when there is no external water that impedes CO_2_ diffusion or cell dehydration that affects the biochemistry of photosynthesis (Dilks & Proctor, 1979; Deltoro *et al*., 1998; Rice *et al*., 2011; Wagner *et al*., 2013; Perera‐Castro *et al*., 2020a,b, 2022b). Thus, the transpiration rate apparently started decreasing before dehydration had an effect on the internal WC of cells, at least if connectivity of tissues and homogeneity of desiccation is assumed. Advanced decreases of the conductance to water before optimum assimilation capacity has been documented previously for mosses (Williams & Flanagan, 1996), but without a clear explanatory reason. The advanced response of transpiration to dehydration also may be related to changes in the meniscus at the numerous air–liquid interfaces between shoot nooks (spaces between leaves and stems) and, therefore, changes in hydrostatic pressure arising from surface tension effect. Changes in leaf position and certain shrinkage of tissues cannot be ruled out. A heterogeneous desiccation of leaves and stems of shoots could also explain the early response of *E* even at WC higher than WC_opt_. According to this plausible explanation, the moment at which shoots achieve WC_opt_ would represent an average between the times at which stems and leaves individually reach WC_opt_, although *E* would start decreasing just before one of these organs reaches its WC_opt_. With or without a mechanism for advanced water control at WC > WC_opt_, our data reveal a persistent disequilibrium between moss water potential and the surrounding atmosphere, challenging the common view that poikilohydric organisms passively track external conditions.

Nonstomatal mechanisms for water control have been shown to be widespread in vascular plants, including C_3_ and C_4_ angiosperms (Márquez *et al*., 2024) and gymnosperms (Cernusak *et al*., 2018). Considering this generality of nonstomatal control in vascular plants, it is plausible that there is a common monophyletic origin rooted on the first land colonizers' ancestors, as has also been suggested for AQPs (Borstlap, 2002; Danielson & Johanson, 2008). In fact, mosses also seem to share the AQP‐dependent biochemical mechanisms that enhance CO_2_ diffusion through the mesophyll in vascular plants (Flexas *et al*., 2006). This is evidenced by the mosses' internal CO_2_ conductance response to temperature (Perera‐Castro *et al*., 2020b), which cannot be explained by physical diffusion alone. Also, mosses present an apparently non‐limiting cell wall thickness, being the relationship between CO_2_ assimilation rates and cell wall thickness reported for vascular plants (Flexas *et al*., 2021) elusive in mosses when big datasets are compiled for them (Perera‐Castro *et al*., 2022a). This lack of correlation between such thick cell walls and *A*_opt_ suggests a biochemical enhancer for CO_2_ diffusion.

Knowing the definitive nature of this water control mechanism would shed light on its evolutionary history. A combination of cell membrane properties (e.g. AQP density and activity), osmotic adjustments within the apoplast, or cell wall channelling (akin to cuticular channels) especially as leaves contort, would be strong candidates for this possibly monophyletic mechanism of nonstomatal water control. Whether this mechanism is not only functionally shared across plant groups but also structurally conserved as an evolutionary pathway remains speculative. A comprehensive study, including data from ferns and lycophytes (currently unavailable to the best of our knowledge), would be required to properly assess this possibility. Conclusively identifying the membrane/cell wall structures responsible for this mechanism in each plant group remains a priority.

### Possible evolutionary constraints to water control in bryophytes

The range of RH_cw,opt_ obtained from the phylogenetically diverse studied species (10 different families) suggests certain interspecific plasticity in the evidenced water control in mosses. The relationships between such RH_cw,opt_ and the pressure–volume curve‐derived parameters (Fig. 4) reflect a possible evolutionary constraint to the efficiency of this nonstomatal water control within mosses. In vascular plants, higher leaf capacitance has been conceived as an evolutionary advantage, since species with this characteristic are able to deal with higher and more variable evaporative demand, concomitant with the highest saturated WC, assimilation rates and lamina hydraulic conductance (Sack *et al*., 2003; Nadal *et al*., 2018; Xiong & Nadal, 2020). Shoot capacitance and elasticity are not associated with assimilation rates in bryophytes (Perera‐Castro *et al*., 2020a), and *A*_opt_ and *E*_opt_ were completely independent in the studied species. Although limiting factors for CO_2_ and H_2_O diffusion seem to be unrelated in bryophytes, having dynamic storage capacity that serves as a buffer to prevent water fluctuations still can be advantageous for mosses with an ‘avoidance’ strategy similar to those proposed by Vitt *et al*. (2014) and Jabłońska *et al*. (2023), but at shoot level. Such an avoidance strategy would be associated with higher water control and, therefore, lower RH_cw,opt_ values.

Mosses with higher capacitance could maximize carbon balance by delaying the decrease in water potential. The assimilation rate, although not maximal, can still be positive in mosses for a relative WC higher than 40% (Perera‐Castro *et al*., 2020a). Furthermore, low dehydration rates are necessary to guarantee the capacity to recover functionality after rehydration in some moss species (Schonbeck & Bewley, 1981; Proctor *et al*., 2007; Cruz de Carvalho *et al*., 2015, 2017; Yuqing *et al*., 2021) and even to prevent desiccation at all, something of vital importance in sensitive species of mosses like *Leucobryum* spp. (Takács *et al*., 1999). The succulence provoked by water storage tissues with flexible cell walls has been considered a drought avoidance mechanism independent of osmotic adjustment in vascular plants (Bartlett *et al*., 2012). Since osmolyte accumulation must imply an energetic and nutritional cost in plant cells, it is consistent that the species with high water control and capacitance also exhibited the lowest osmotic adjustment.

The avoidance/spender strategy described by RH_cw,opt_ and the pressure–volume‐derived parameter is consistent with the ecophysiology already known for some of the studied species. *Hygrohypnum* spp. is commonly known as the brook moss for its high affinity to aquatic environments (Glime, 1994), habitats where the control of water loss is not required at all (RH_cw,opt_ = 94%). Some Polytrichaceae species included in this study (*Atrichum undulatum*, *P. commune*, and *P. aloides*) also presented values of RH_cw,opt_ more associated with a spender strategy (RH_cw,opt_ < 93%), probably related to the advanced hydraulic system described for this group of bryophytes (Brodribb *et al*., 2020). Although epicuticular wax has been described in some Polytrichaceae species (with the exception of *Atrichum* spp., among others), the presence of a thin and discontinuous water repellent surface has been more closely associated with protection against water‐logging than with control of water loss (Clayton‐Greene *et al*., 1985), something that has a clear advantage in enhancing CO_2_ diffusion (Proctor, 2005). Our data are consistent with this interpretation, since all Polytrichaceae examined species exhibited limited control over water loss regardless of the presence or absence of wax.

The opposite strategy can be observed for *L. albidum*, whose desiccation sensitivity described above maybe can only be compatible with specialized cells that maximize water storage (dead hyaline cells) and a certain control of water loss both at hydrated state (RH_cw,opt_ = 71.9%) and during dehydration (*r*_H2O_ at TLP = 15.3 ± 2.4 MPa m^2^ s mmol^−1^). *Pohlia nutans* and *C. purpureus*, two cosmopolitan species inhabiting in a wide range of substrates (Ochyra *et al*., 2008), also presented significant water control during optimum conditions (RH_cw,opt_ of 59.4 and 72.6%, respectively) but their behavior differed during dehydration, when *r*_H2O_ remained among the lowest for *C. purpureus* (2.7 ± 0.2 MPa m^2^ s mmol^−1^) contrary to *P. nutans* (*r*_H2O_ = 9.6 ± 3.1 MPa m^2^ s mmol^−1^). Although this classification may overlook species‐specific nuances, the emerging pattern suggests that some form of water control is deeply embedded in the biology of mosses across environments. The final water relations of mosses must therefore result from the combined effects of the storage capacity of external capillary water (Dilks & Proctor, 1979; Proctor *et al*., 1998), the storage capacity of internal water at the shoot level (Proctor & Tuba, 2002; Perera‐Castro *et al*., 2020a) and membrane/cell wall resistance to water loss evidenced in our study.

### Conclusions

Our results indicate that mosses exhibit a nonstomatal control of water loss that has not been previously reported. The variation in transpiration and relative humidity of the cell wall, along with the differences between cell wall and cytoplasm water potential, support the idea of a nonstomatal water control mechanism in mosses. The magnitude of the decrease in cell wall water potential correlated with traits associated with an avoidance strategy for coping with fluctuations in water availability in mosses – including cytosolic water storage, high tissue capacitance and elasticity, and a lack of osmotic adjustment. This mechanism of water loss control through cell membranes and/or cell walls is possibly derived from common ancestral traits shared with vascular plants, related to the unsaturated conditions of internal airspaces. The nature of this mechanism is unknown. Our findings reveal that mosses, due to their simple structure and the absence of stomata in the gametophyte, provide key insights into the nature and evolutionary significance of this mechanism.

## Competing interests

None declared.

## Author contributions

AVP‐C, FAB, DAM, and DTH contributed to the design of the study and the interpretation of the results; AVP‐C performed data collection, analysis, and writing of the original draft. All authors contributed to the final version of the manuscript.

## Disclaimer

The New Phytologist Foundation remains neutral with regard to jurisdictional claims in maps and in any institutional affiliations.

## Supporting information


**Fig. S1** Custom‐made moss cuvette.
**Fig. S2** Relationship between moss shrinkage and water content during dehydration for each species.
**Fig. S3** Graphic illustration of the pressure–volume derived parameters and their calculation.
**Fig. S4** Sensitivity analysis of relative humidity of the cell wall in response to variations of boundary layer conductance.
**Fig. S5** Sensitivity analysis of relative humidity of the cell wall in response to variations of moss temperature estimated by energy balance.
**Fig. S6** Sensitivity analysis of relative humidity of the cell wall in response to moss absorbance of shorter wavelength.
**Fig. S7** Pressure–volume curves obtained for all studied species.
**Fig. S8** Response to dehydration of net CO_2_ assimilation and transpiration for detached leaves and shoots.
**Fig. S9** Differences between detached leaves and shoots in their transpiration, cell wall humidity, and time for dehydration.
**Fig. S10** Relationship between at cell wall at optimum water content and cell wall vapor potential.
**Fig. S11** Relationship between at cell wall at optimum water content and saturated water content of cytosol and apoplast per unit shoot area.
**Fig. S12** Relationship between cytoplasmic water potential and cell wall water vapor potential and resistance of cell wall and plasmatic membranes to water for detached leaves and shoots.
**Methods S1** Analysis to characterize the relationship between shoot area shrinkage and water content.
**Methods S2** Analysis to characterize microscopy and anatomy.

**Table S1** Dataset generated in this study for all species.
Please note: Wiley is not responsible for the content or functionality of any Supporting Information supplied by the authors. Any queries (other than missing material) should be directed to the *New Phytologist* Central Office.

## Data Availability

All data supporting the findings of this study are available from the *CORA Repositori de Dades de Recerca* of the University of Balearic Islands (doi: 10.34810/data3170) or within its Table S1 published online.
