# Supplementary material for: Evidence for regulation of transpiration in nonstomatal plants: insights from bryophyte gametophytes
Source: New Phytol. 2026 Apr 24;252(1):54–68. doi: 10.1111/nph.71206 (PMC13539947; doi:10.1111/nph.71206)
Supplement: Supplementary file 1 [file NPH-252-54-s001.pdf]

New Phytologist

***New Phytologist* Supporting Information**

Article title: Evidence for regulation of transpiration in non-stomatal plants: insights from bryophyte gametophytes
Authors: Alicia V. Perera-Castro, Diego A. Márquez, Florian A. Busch, David T. Hanson
Article acceptance date: 27 March 2026

The following Supporting Information is available for this article:

Fig. S1 Custom-made moss cuvette.

Fig. S2 Relationship between moss shrinkage and water content during dehydration for each species.

Fig. S3 Graphic illustration of the pressure-volume derived parameters and their calculation.

Fig. S4 Sensitivity analysis of relative humidity of the cell wall at optimum water content in response to variations of boundary layer conductance.

Fig. S5 Sensitivity analysis of relative humidity of the cell wall at optimum water content in response to variations of moss temperature estimated by energy balance.

Fig. S6 Sensitivity analysis of relative humidity of the cell wall at optimum water content in response to moss absorbance of shorter wavelength.

Fig. S7 Pressure-volume curves obtained for all studied species.

Fig. S8 Response to dehydration of net CO_2 assimilation and transpiration for detached leaves and shoots.

Fig. S9 Differences between detached leaves and shoots in their transpiration, cell wall humidity and time for dehydration.

Fig. S10 Relationship between relative humidity at cell wall at optimum water content and cell wall vapor potential.

Fig. S11 Relationship between relative humidity at cell wall at optimum water content and saturated water content of cytosol and apoplast per unit shoot area.

Fig. S12 Relationship between cytoplasmic water potential and cell wall water vapor potential and resistance of cell wall and plasmatic membranes to water for detached leaves and shoots.

Method S1 Analysis to characterize the relationship between shoot area shrinkage and water content.

Method S2 Analysis to characterize microscopy and anatomy.

Table S1 Dataset generated in this study for all species (submitted separately).

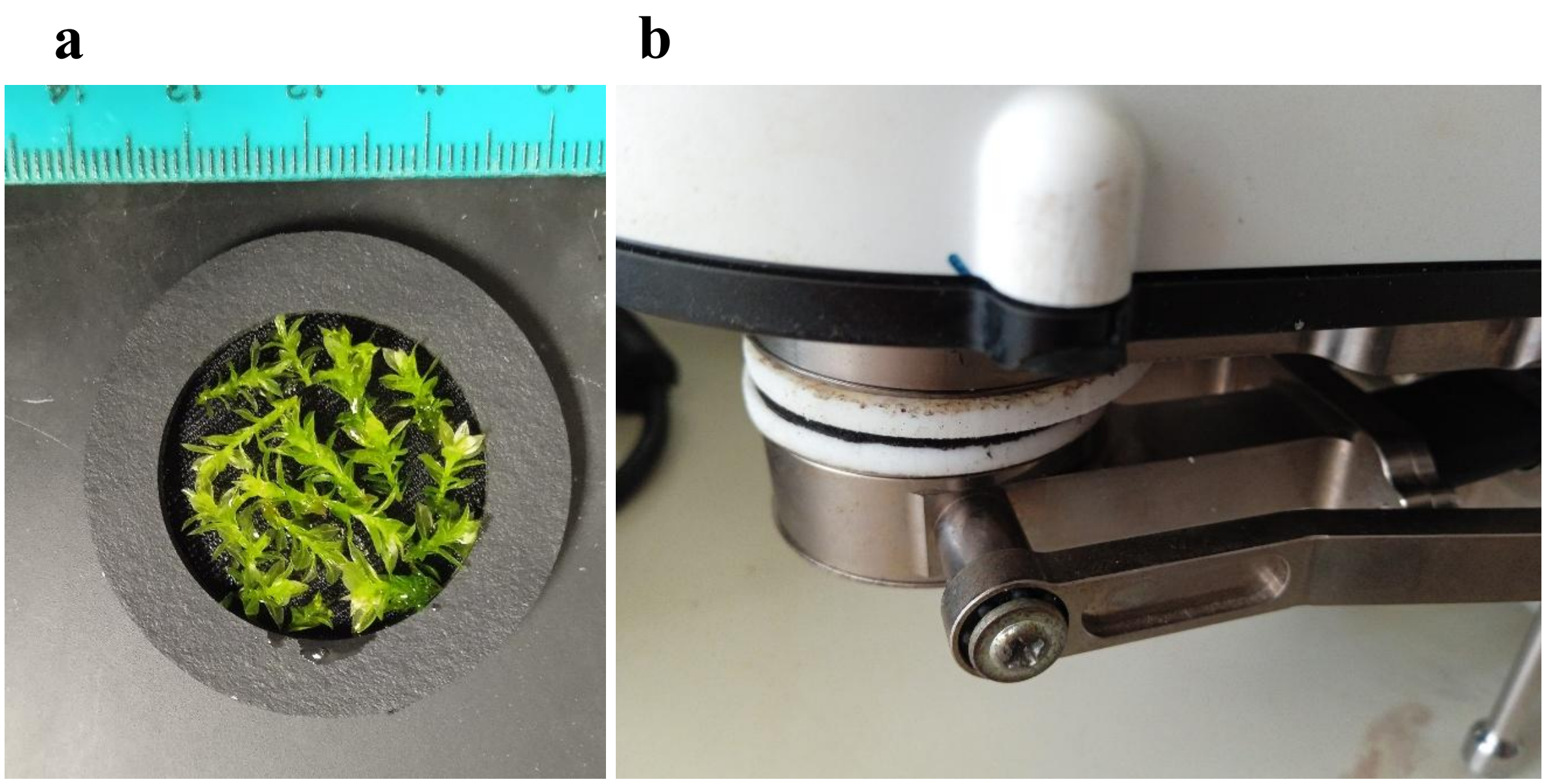

Fig. S1 (a) Custom-made moss cuvette consisting of a LI-6800 gasket affixed to a piece of thin polyester stocking fabric. Soil and brown tissues as well as excessive interstitial water were removed from moss. Mosses were placed on the fabric with shoot overlap minimized. **(b)** The cuvette containing the sample was placed inside the LI-6800 chamber, ensuring that the cuvette and chamber gaskets were aligned.

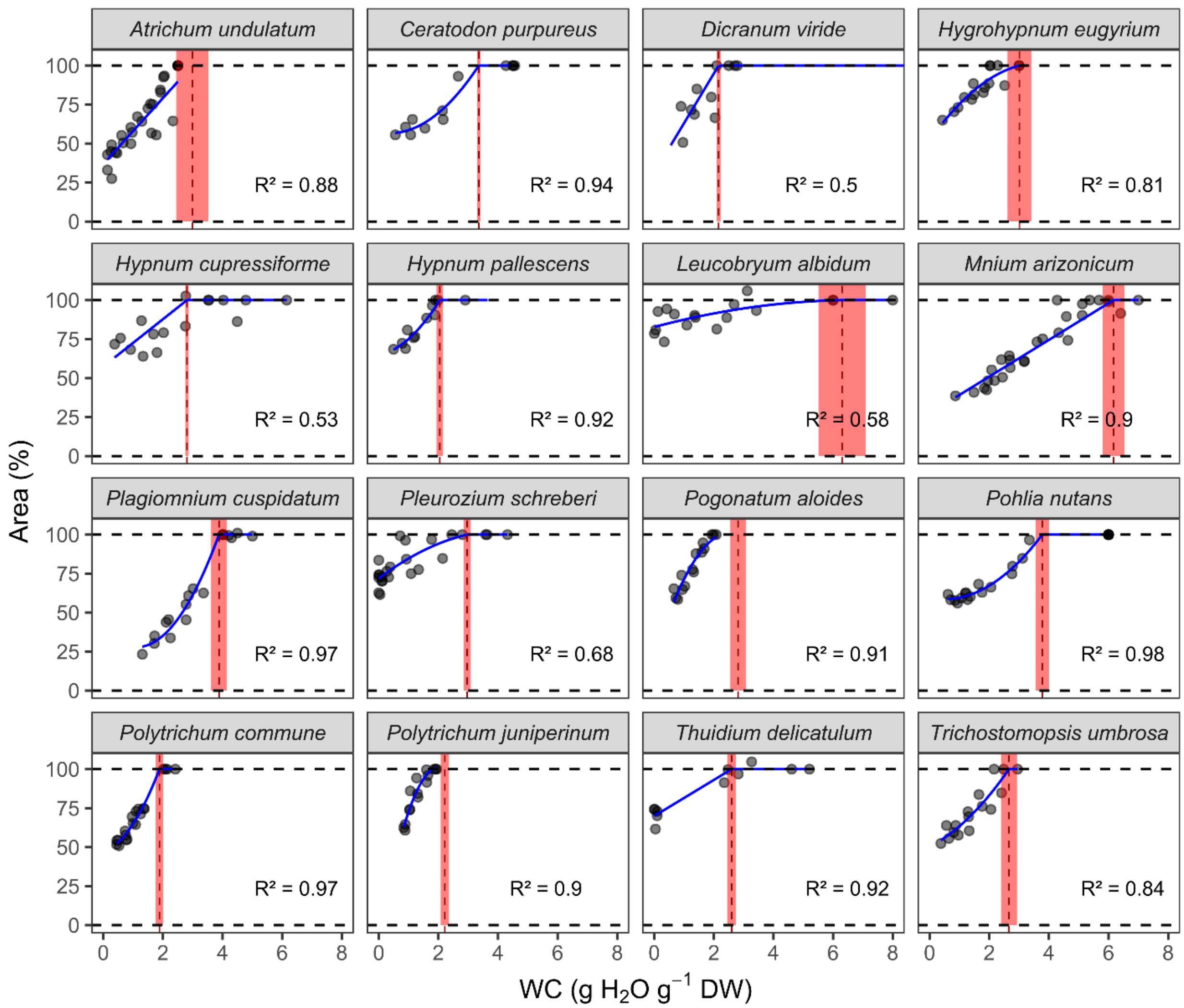

Fig. S2 Relationship between projected area (% of initial area) and water content (WC) during dehydration for each species. For each species, the best-fitting model (linear or quadratic, Method S1) was selected based on the coefficient of determination (R^2). Models were constrained to pass through the species-specific optimal WC (WC_{opt}, red rectangle ±SD) where projected area was assumed to be 100%. Observations to the right of WC_{opt} (hydrated tissues) were assumed to maintain full area. Blue lines represent model predictions. Dashed horizontal and vertical lines indicate 100% area and WC_{opt}, respectively.

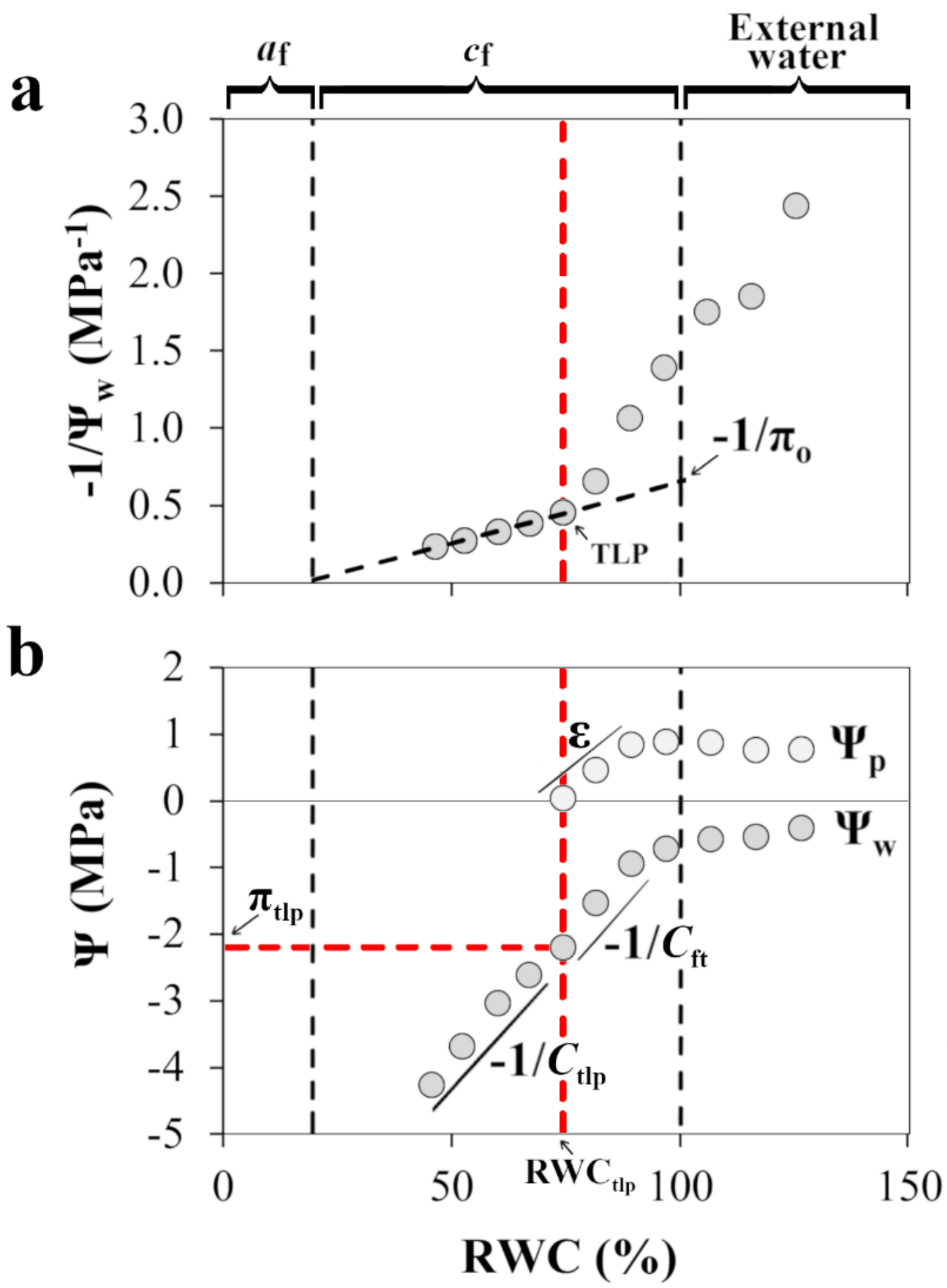

Fig. S3 (a) Pressure-volume curve for a bryophyte illustrating the relationship between the inverse of cell water potential (Ψ_w) and relative water content (RWC). **(b)** Variation of Ψ_w and pressure potential (Ψ_p) along RWC. During sample dehydration (decrease of RWC), the point at which the relationship between -1/Ψ_w begins to be linear is called the turgor loss point (TLP). The relative water content and water potential at this point are referred to as RWC_{tlp} and π_{tlp}, respectively (follow red lines). The x-axis intercept of the linear function of the pressure–volume curve indicates the point at which the sample has lost all of its cytosolic water fraction (c_f) and the apoplastic fraction (a_f) begins. Extrapolating this linear function to x = 100% allows us to determine the negative inverse of the osmotic potential at full turgor (π_o). Capacitance at full turgor (C_{ft}) and at turgor loss point (C_{tlp}) was determined from the relationship between RWC and RWC above and below turgor loss point, respectively. Bulk modulus of elasticity (ε) was calculated as the slope Ψ_p versus RWC. Data measured for a sample of *Thuidium tamariscinum* collected in Mallorca in 2018.

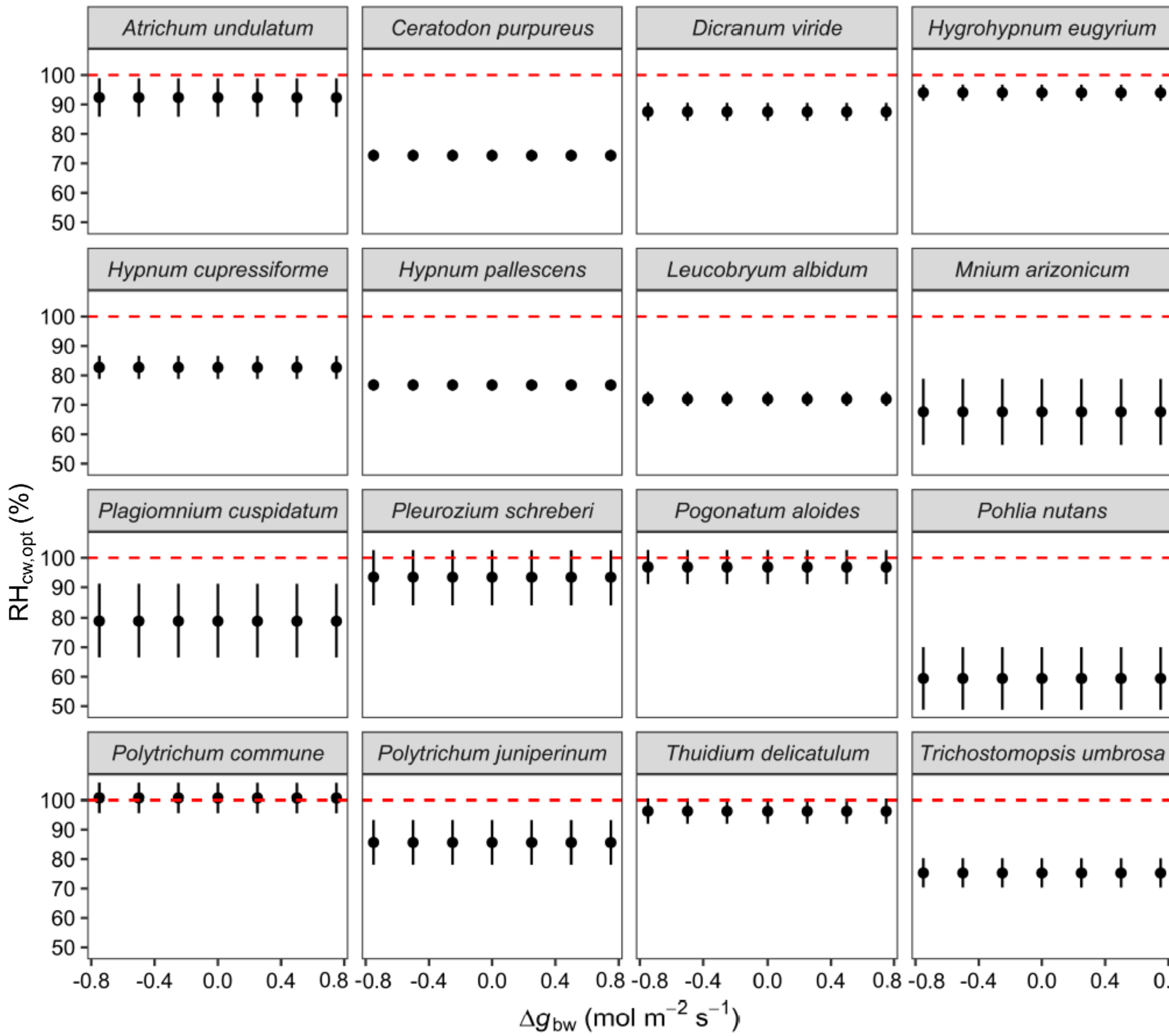

Fig. S4 Sensitivity analysis of relative humidity of the cell wall at optimum water content ($RH_{cw,opt}$, mean ±SD) of all studied species in response to variations of boundary layer conductance (Δg_{bw}) of ± 0.75 mol m^{-2} s^{-1} relative to the original recorded values (1.7-2 mol m^{-2} s^{-1}). Red line indicates $RH_{cw,opt}$ of 100%.

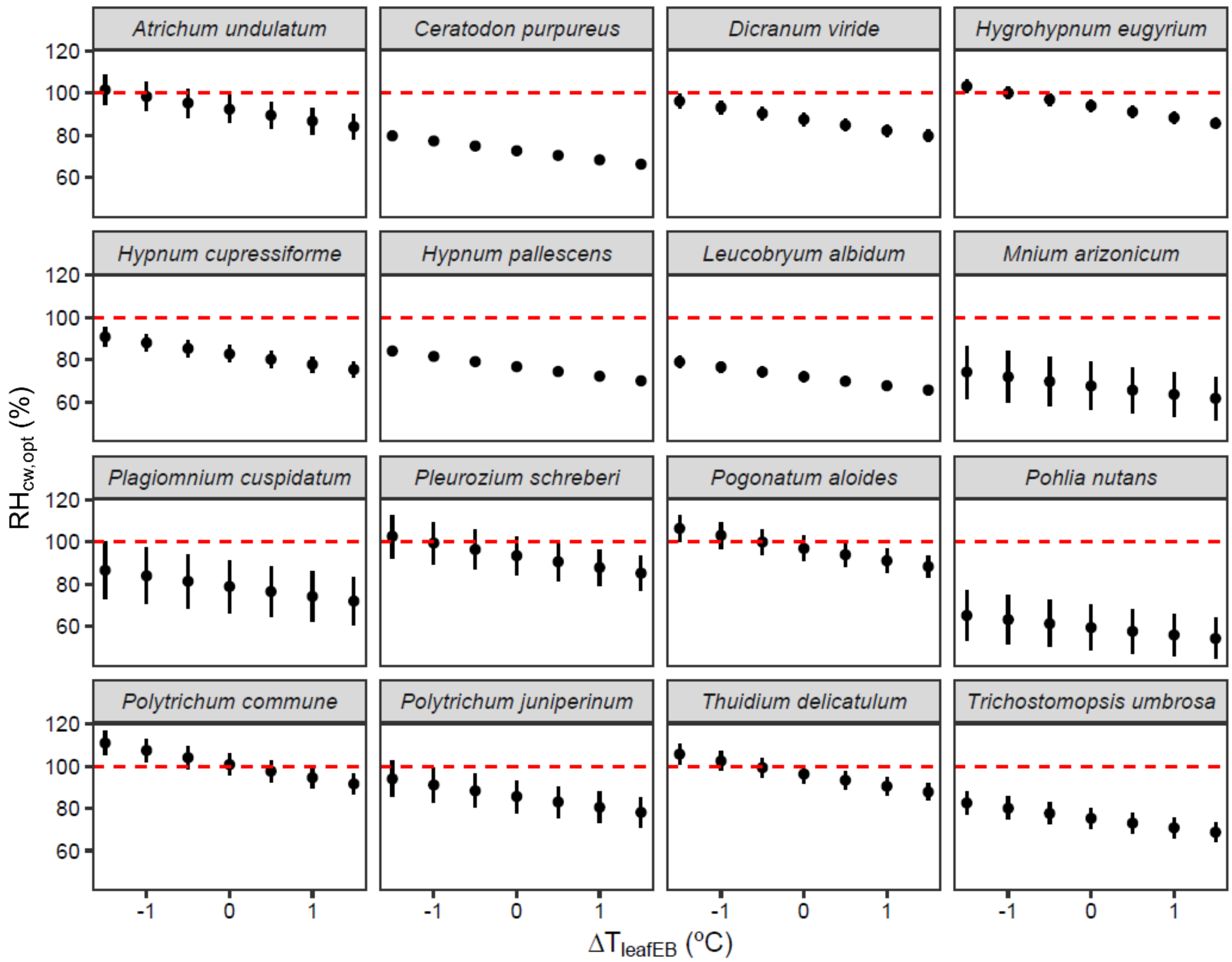

Fig. S5 Sensitivity analysis of relative humidity of the cell wall at optimum water content ($RH_{cw,opt}$, mean ±SD) of all studied species in response to variations of moss temperature estimated by energy balance (ΔT_{leafEB}) of ± 1.5ºC relative to the original recorded values (17.7-21.5ºC). Red line indicates $RH_{cw,opt}$ of 100%.

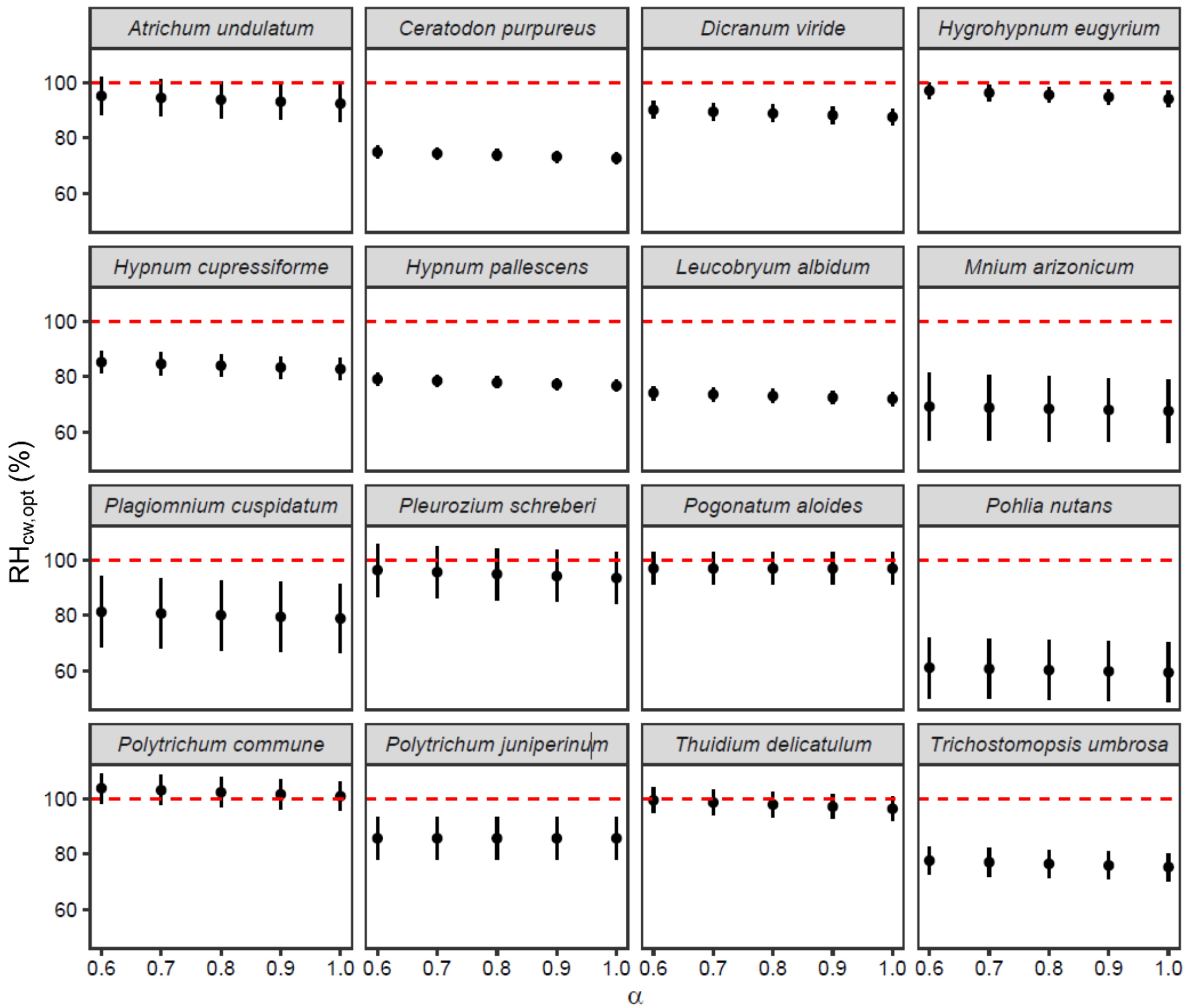

Fig. S6 Sensitivity analysis of relative humidity of the cell wall at optimum water content ($RH_{cw,opt}$, mean ±SD) of all studied species in response to moss absorbance of shorter wavelength (α). An ample range of α between 0.6 and 1 was assumed. Red line indicates $RH_{cw,opt}$ of 100%.

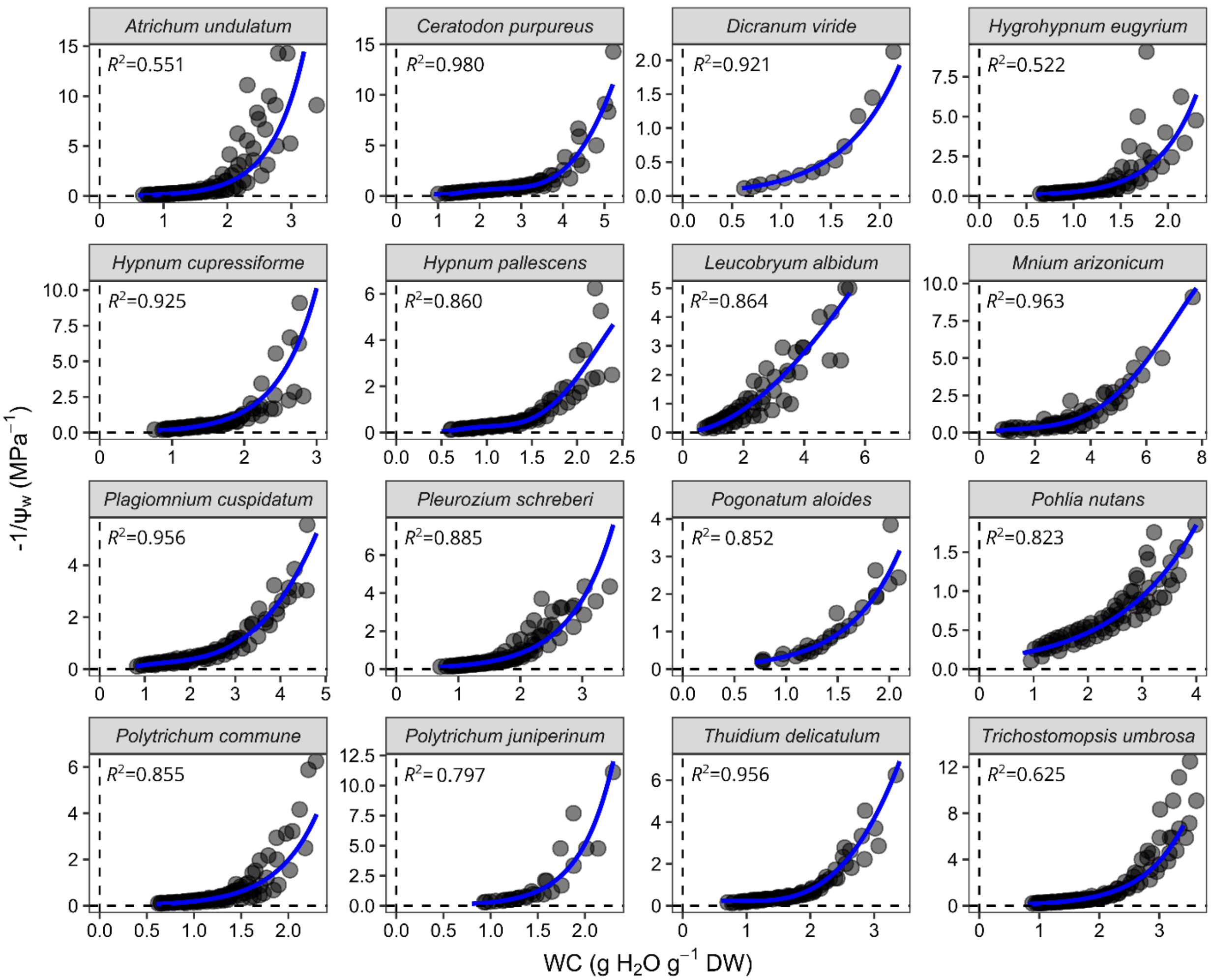

Fig. S7 Pressure-volume curves obtained for all studied species. Blue line indicates the polynomial or exponential adjustment of the relationship between negative inverse of water potential ($-1/\Psi_w$) and water content (WC).

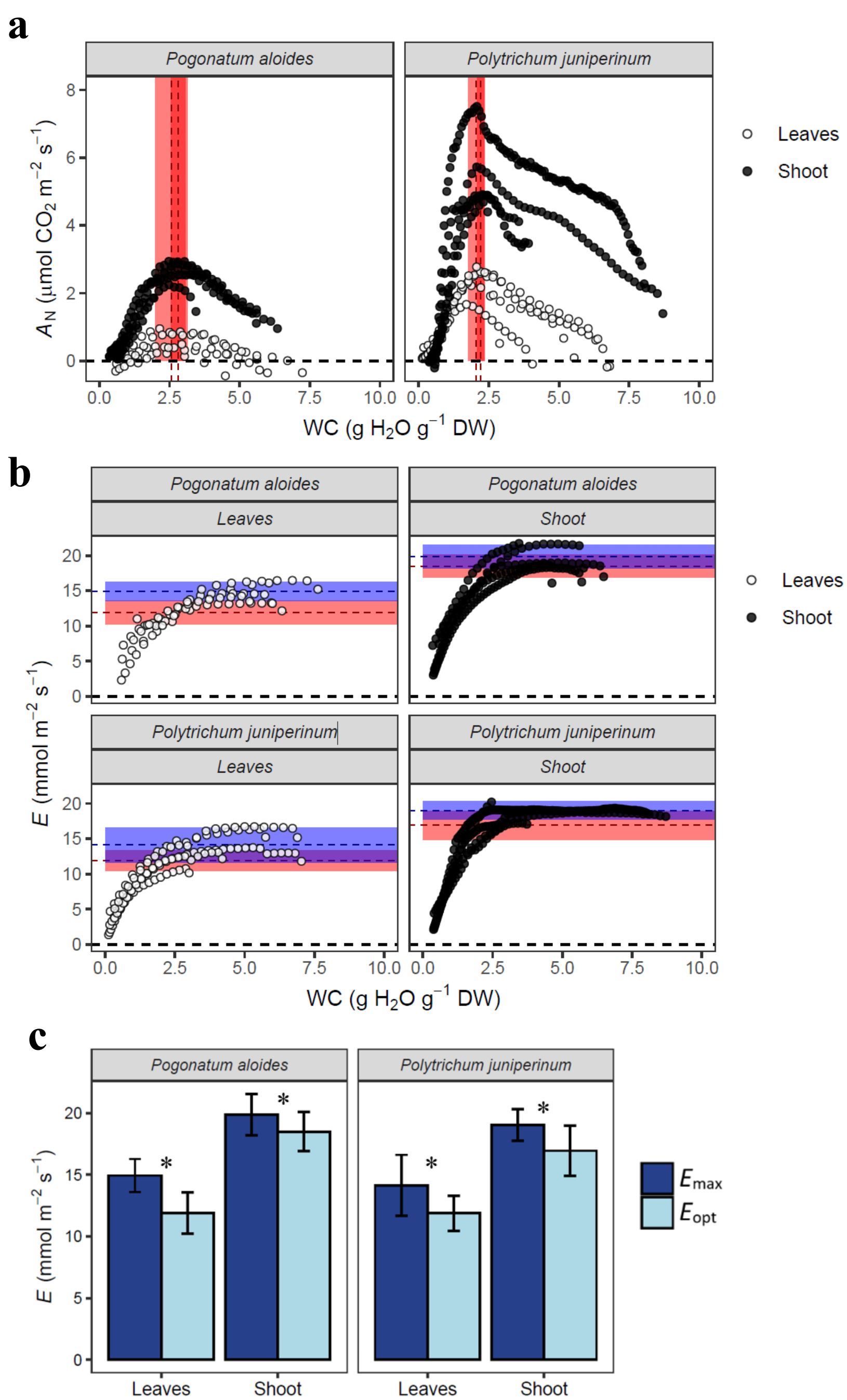

Fig. S8 Response to dehydration of **(a)** net CO_2 assimilation rate (A_N) and **(b)** transpiration rate (E) for detached leaves and shoots of *Pogonatum aloides* and *Polytrichum juniperinum*. Red vertical lines indicate the average water content (WC) at which maximum A_N (A_{opt}) is found (WC_{opt}). Red and blue horizontal lines indicate averaged transpiration rates at WC_{opt} (E_{opt}) and maximum transpiration rates (E_{max}), respectively. Shaded areas indicate ±SD of the respective parameters. **(c)** Differences between E_{max} and E_{opt} of shoots and leaves of both species. Bars denotate mean ±SD. Asterisks indicate significant differences by paired *t*-test.

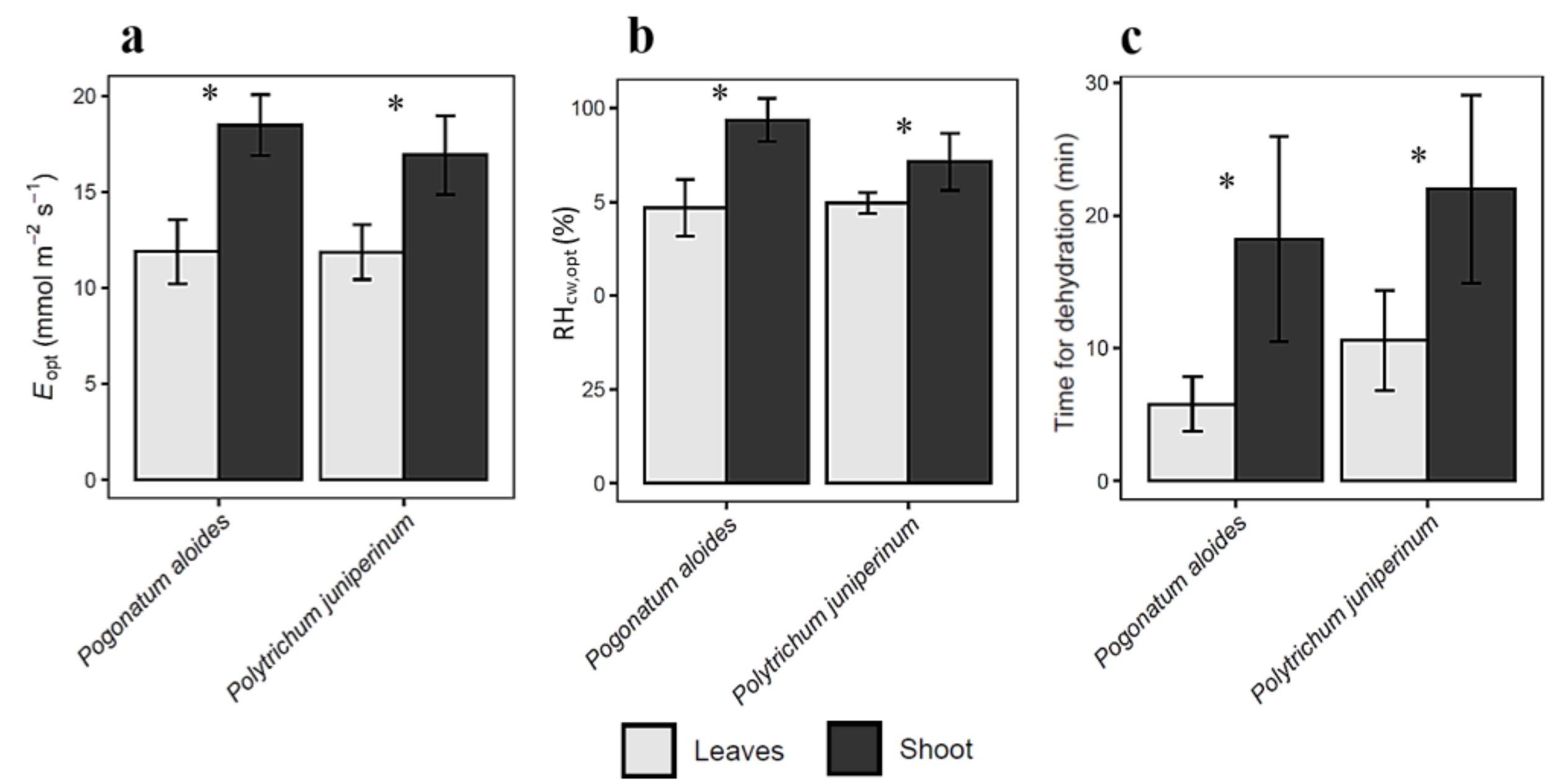

Fig. S9 Differences between leaves and shoots of *Pogonatum aloides* and *Polytrichum juniperinum* in the **(a)** transpiration rate at the maximum net CO_2 assimilation (E_{opt}), **(b)** relative humidity at the cell wall in optimum water content ($RH_{cw,opt}$) and (c) time for dehydration (from maximum CO_2 assimilation to cero net CO_2 fluxes in the dehydration curves). Bars denotate mean ±SD. Asterisks indicate significant differences by *t*-test.

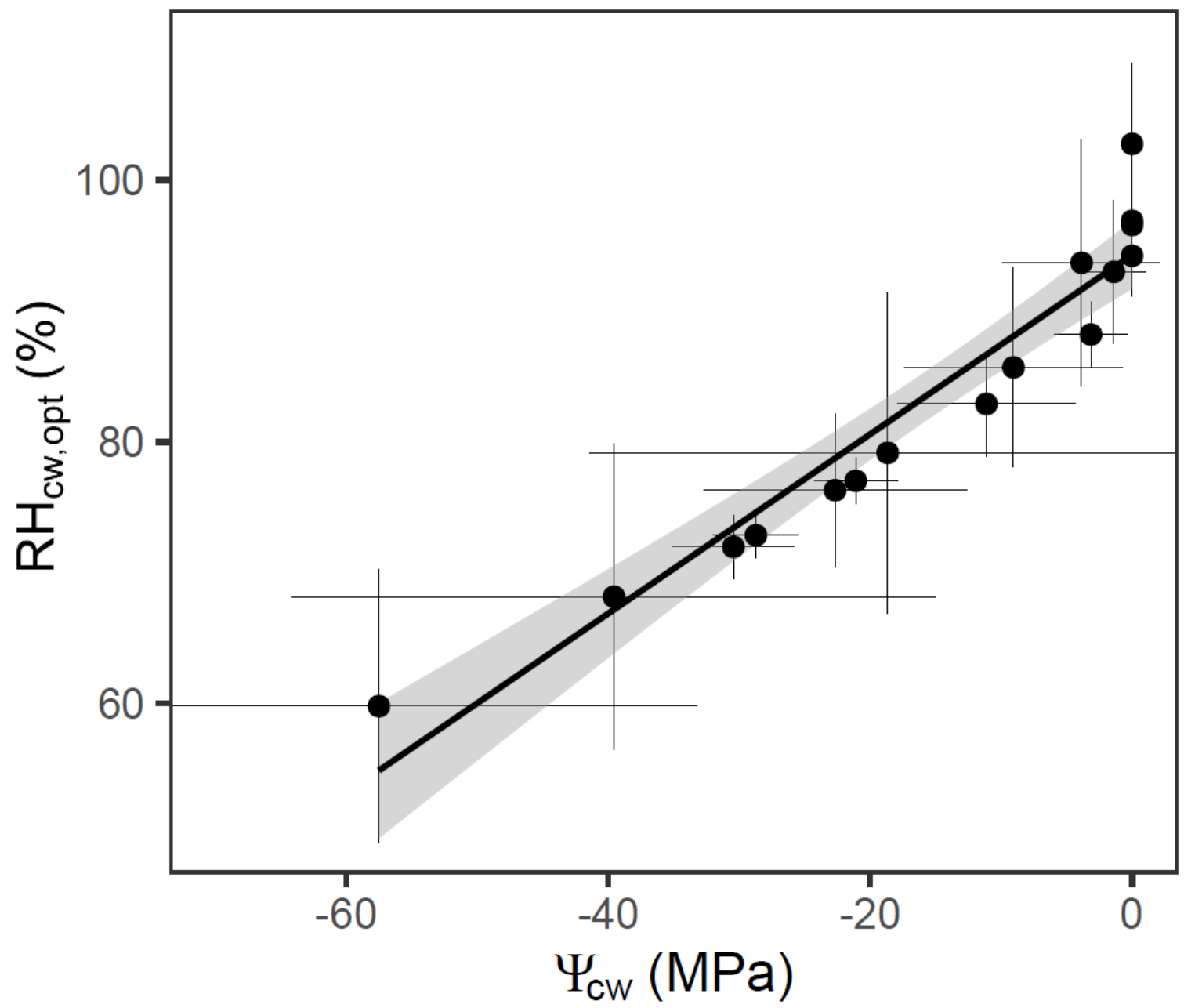

Fig. S10 Relationship between relative humidity at cell wall at optimum water content ($RH_{cw,opt}$) and the equivalent cell wall water vapor potential (Ψ_{cw}) obtained from Eq 4 of the main manuscript. Points represent the mean ±SD for each species.

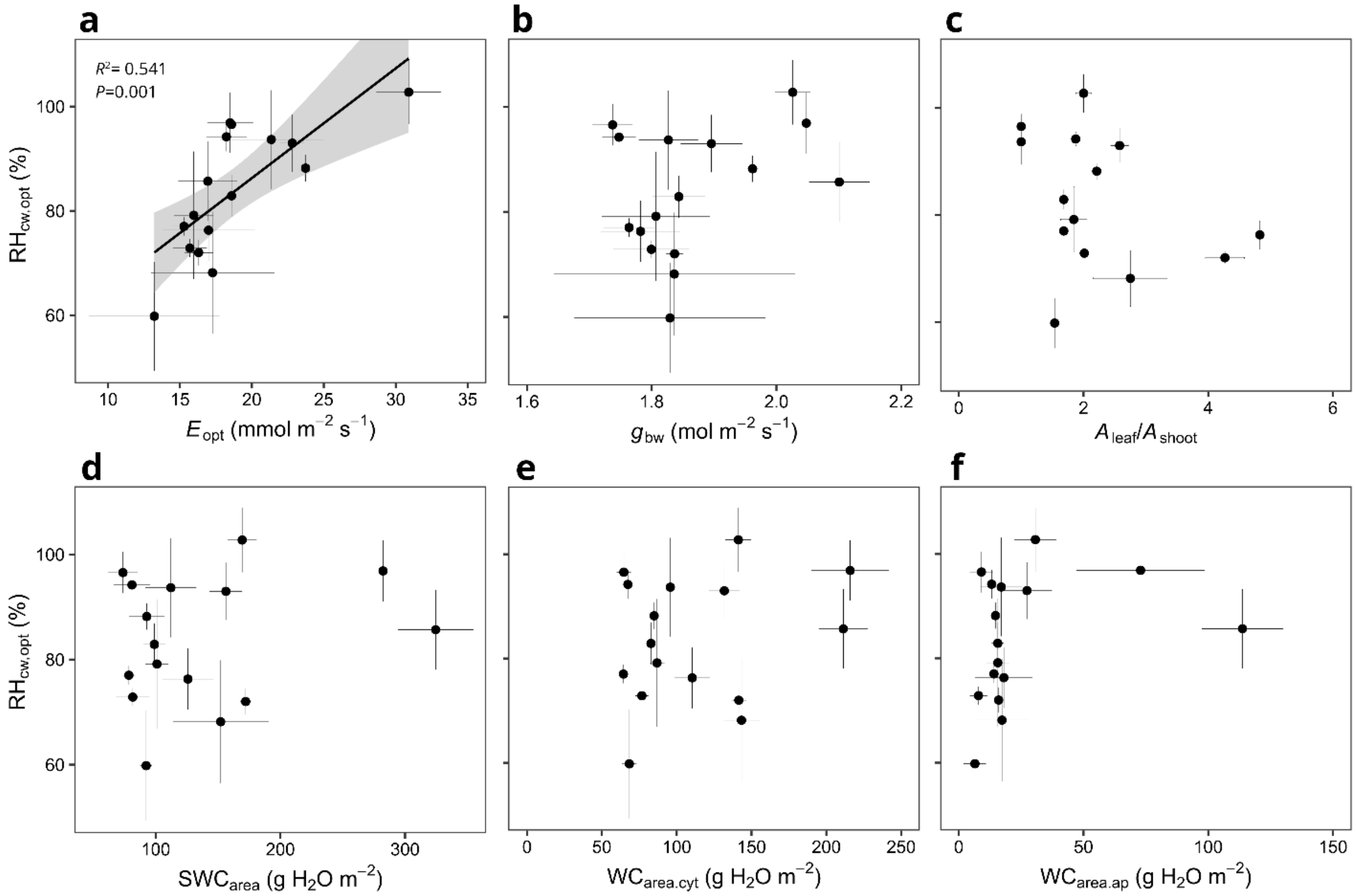

Fig. S11 Relationship between relative humidity at the cell wall at optimum water content ($RH_{cw,opt}$) and (a) transpiration rate at optimum water content (E_{opt}), (b) conductance of the boundary layer to the diffusion of water (g_{bw}), (c) ratio of leaf area per shoot area (A_{leaf}/A_{shoot}), (d) saturated water content per unit shoot area (SWC_{area}), saturated water content of cytosol (e) and apoplast (f) per unit shoot area ($WC_{area.cyt}$ and $WC_{area.ap}$, respectively). Points represent the mean ±SD for each species (n=3-5, n=1-5 for A_{leaf}/A_{shoot}).

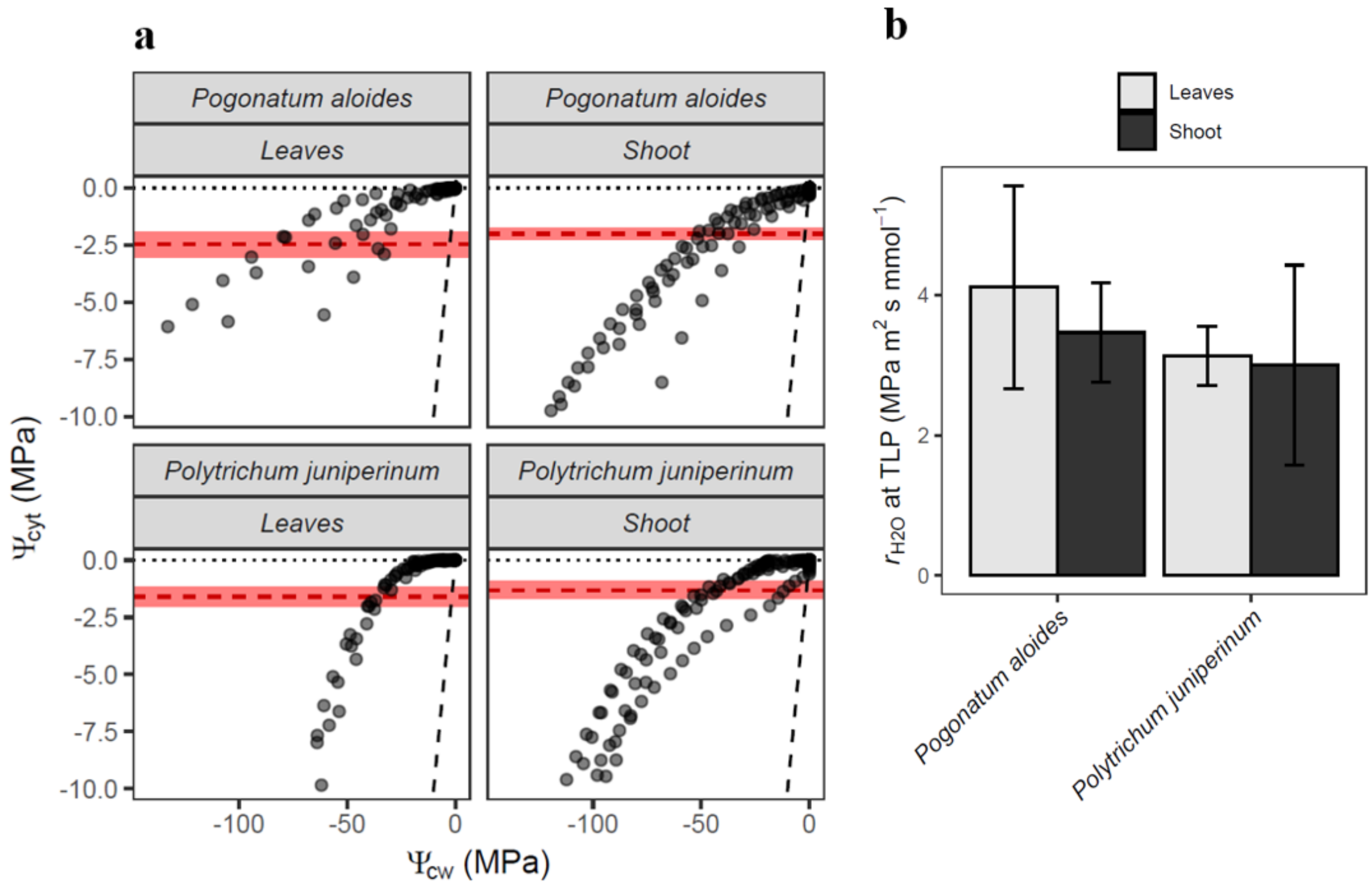

Fig. S12 (a) Relationship between cytoplasmic water potential (Ψ_{cyt}) and cell wall water vapor potential (Ψ_{cw}) for the dehydration curves of leaves and shoots of *Pogonatum aloides* and *Polytrichum juniperinum*. Ψ_{cyt} was calculated from the WC at any moment of the dehydration curve by using the relationship between $-1/\Psi_w$ and WC of pressure volume curves (Fig. S7) and assuming Ψ_w=Ψ_{cyt}. The horizontal dashed red line represents the averaged water potential at the turgor loss point (π_{tlp}). Shaded areas indicate ±SD. The diagonal dashed black line indicates a 1:1 relationship. **(b)** Equivalence between leaves and shoots of both species in resistance of cell wall/plasmatic membrane to water movement (r_{H2O}) at turgor loss point (TLP).

Methods S1: corrections for area shrinkage

To characterize the relationship between shoot area shrinkage and water content (WC), we fitted two types of regression models for each species: a linear model and a quadratic (second-order polynomial) model. Both models were constrained to pass through the species-specific optimal water content value (WC_{opt}), defined as the mean WC value at which area loss was assumed to be minimal (i.e., Area = 100%). For each species, only observations with WC values less than or equal to WC_{opt} were retained for model fitting, under the assumption that tissue function remains unimpaired above this threshold. The linear model was specified as follows:

$$Area\ (\%) = \beta_1 \cdot \left(WC - WC_{opt}\right) + 100$$

where β1 represents the slope of the response curve prior to the onset of area loss.

The quadratic model was defined as:

$$Area\ (\%) = \beta_1 \cdot \left(WC - WC_{opt}\right) + \beta_2 \cdot (WC - WC_{opt})^2 + 100$$

where β1 and β2 correspond to the linear and quadratic components, respectively. In both cases, the models were fitted without intercepts to enforce the condition Area = 100% at WC = WC_{opt}. Predictions were generated across the observed WC range for each species. For WC values greater than WC_{opt}, the projected shoot area was fixed at 100%, reflecting the assumption that no area loss occurs beyond the optimal hydration point. Model performance was evaluated using the coefficient of determination (R^2), and all gas exchange-derived parameters were based on the corrected area considering its shrinkage along the dehydration curve. All analyses were performed in R (version 4.2.3) using the *lm*() function and the *tidyverse* package for data manipulation and visualization.

Methods S2: microscopy and anatomy analysis

Three shoots per species were selected for microscopy analysis, except for *Polytrichum juniperinum* and *Pogonatum aloides*. Sample manipulation and excision of leaves (phyllids) were manually done under a stereoscopic microscope. Images of cross sections and leaf surfaces were obtained with a camera (CMOS USB 3.0 10MP Camera) attached to a light microscope (Leica DM6 B Microscope). Images were obtained from the central region of the leaves, excluding the midrib in surface views. Images taken with a magnification of 400X were analysed with ImageJ for obtaining the cell wall thickness (T_{cw}) from the cross-section images, and cell areas (A_{cell}) from leaf surface images. In the case of Polytrichaceae species, A_{cell} was obtained from the surface of a detached lamella. Each replicate (*n*=3) represents the average of 3 to 20 measurements that were taken for each parameter and sample whenever possible. As an indirect measurement of the total exposure surface area, measurements of the total leaf area per shoot area (A_{leaf}/A_{shoot}) were done by detaching all leaves of a shoot (*n*=1-5). A stereoscopic microscope was used in some species. For *Pleurozium schreberi* and *Thuidium delicatulum*, A_{leaf}/A_{shoot} was assumed to be close to 1, due to the tiny size of their dispersed leaves.
